# Supplementary material for: Deciphering splicing heterogeneity at single-cell resolution by SCSES
Source: Nat Commun. 2025 Oct 27;16:9459. doi: 10.1038/s41467-025-64517-5 (PMC12559242; doi:10.1038/s41467-025-64517-5)
Supplement: Supplementary file 1 — Supplementary Information [file 41467_2025_64517_MOESM1_ESM.pdf]

Supplementary Information for

Deciphering splicing heterogeneity at single-cell resolution by SCSES

Xiao Wen[1,2,*], Xuan Lv[1,2,3,*], Dan Guo[1,2], Nan Han[1,2,3], Lei Zhou[1,2,3], Peizhuo Wang[4], Zhaoqi Liu[1,2,#]

[1]Computation Biology Department, China National Center for Bioinformation, Beijing 100101, China.

[2]Beijing Institute of Genomics, Chinese Academy of Sciences, Beijing 100101, China.

[3]University of Chinese Academy of Sciences, Beijing 100049, China

[4]School of Life Science and Technology, Xidian University, 710071, Xi'an, Shaanxi, China

*These authors contributed equally

#Correspondence: liuzq@big.ac.cn (Z.L.)

Supplementary Note 1. Sequence feature for different splicing types

For SE events, four types features are used to calculating event similarities, including length features, motif features, conservation features and k-mer features. As shown in Supplementary Fig. 37, an SE event is associated with five regions: the first exon (C1), the first intron (I1), the alternative exon (A), the last intron (I2), and the last exon (C2). The length features include the length of region C1, C2 and A, as well as the length ratio of A/C1, A/C2 and C1/C2. The motif features include the scores of average position weight matrix (PWM) from regions near 3'/5' splicing site of I1 and I2. The nearby regions are defined between 16nt upstream to 4nt downstream for 3' splicing site, and between 4nt upstream to 6nt downstream for 5' splicing site. The conservation features include the conservation scores of regions C1, A, and C2, as well as the regions near 3'/5' splicing site of region I1 and I2, calculated by phastCons[1]. The k-mer features include the 1-3mers of region C1, C2, nearby regions of I1 3' splicing stie and I2 5' splicing site, 1-2mers of nearby region of I1 5' splicing site and I2 3' splicing site, and 1-4mers of region A. The features of an MXE event are the combination features of two related SE events (Supplementary Fig. 37). Features for A3SS, A5SS and RI events are defined similarly (Supplementary Data 11, Supplementary Fig. 37). Furthermore, we define an adenine ratio feature to account for alternative branch point selection for A3SS, A5SS, and RI events. Specifically, for an alternative region in an event (Supplementary Fig. 37), we divide the region into 100 bins with equal length, and calculate the cumulative adenine ratio for each bin as the adenine ratio features.

Supplementary Note 2. Datasets for algorithm evaluation

Artificial synthetic dataset generation

Spanki simulator was used to synthesize the artificial datasets for evaluation. Spanki can generate a forge sequencing file, given the reference bam file and expression values of isoforms. Here we used the bulk sequencing results of four cell lines (HCT116, HCT1954, HepG2, HL-60) as references, and generated cell populations for each cell line. We propose a pipeline to simulate the isoform expression and scRNA-seq technique noise in each cell, as

well as remaining the splicing regulation relationships and splicing difference among cell lines:

(1). Identify isoforms and quantify their expressions with reads assigned per kilobase (RPK) in bulk sequencing cell line datasets by Cufflinks[2];

(2). Identify all the annotated SE events by SUPPA[3], and quantify their splicing levels in each cell line;

(3). Identify the marker events for each cell line, whose PSI values are larger 0.1 than other cell lines, and denote as ME; the genes associated with these events are denoted as $MG = \{mg_{HCT116}, mg_{HCT1954}, mg_{HepG2}, mg_{HL-60}\}$ for marker genes;

(4). Create a candidate gene set consisted of splicing factors SF, genes for regression fine-tuning FT, genes in MG and other genes OG;

(5). Create an event pool consisted of 1000 events randomly, where there are 30% events from ME, and all events from SF and FT; the genes associated with the event pool are denoted as PG;

(6). For each isoform iso of PG in cell line cl, we use the RPK value $RPK_{bulk}(iso, cl)$ from bulk sample in cl as the standard expression level, and denote $rt > 1$ as the scaling factor to simulate the low coverage of scRNA-seq data; the average expression of iso in cl single cells is computed as $RPK_{sc}(iso, cl) = RPK_{bulk}(iso, cl)/rt$;

(7). The dropout probability is correlate with gene expression in a logistical form[4]. We define τ as the expression where dropout probability is 0.5, and the dropout probability of 0 is $1 - 10^{-\kappa}$. The dropout rate of gene g can be calculated by:

$$P_{dropout}(g, cl) = 1 - \frac{1}{1+e^{-\eta(RP\ \ sc(g.cl)-\kappa)}}, \tag{S1}$$

where $\eta\tau = \ln(10^{\kappa} - 1)$; in this study, we set $\kappa = 3$; next, for cell c we sample the dropout with Bernoulli distribution, where $DR_c(g, cl) \sim B\left(M, P_{dropout}(g, cl)\right)$;

(8). We use the Gaussian distribution and gamma distribution to emulate the expression value with noise in the dataset. There is a linear relationship between the mean and the variance of isoform log-transformed expression, namely $\ln\left(Var_{iso,cl} + 1\right) = \gamma \ln(RPK_{sc}(iso, cl) + 1)$. It can be transformed as $Var_{iso,cl} = (RPK_{sc}(iso, cl) + 1)^{\gamma} - 1$, where γ can be seen as the noise level. Hence, for gamma distribution $Gamma(\alpha, \beta)$, we can obtain

parameters by $\alpha = \frac{RPK_{sc}^2(iso,cl)}{Var_{iso,cl}}$ and $\beta = \frac{Var_{iso,cl}}{RPK_{sc}(iso,cl)}$. For Gaussian distribution $Norm(\mu, \sigma^2)$, $\mu = RPK_{sc}(iso,cl)$ and $\sigma^2 = Var_{iso,cl}$.

(9). Combining the all the parameters, we can obtain the expression of isoform in a cell c by:

$$\boldsymbol{RPK_c} \sim \begin{cases} 0 & if\ DR_c(g,cl) = 1 \\ Norm(\mu,\sigma^2) & if\ RPK_{sc}(iso,cl) < Var_{iso,cl}\ and\ DR_c(g,cl) = 0 \\ Gamma(\alpha,\beta) & others \end{cases} \quad (S2)$$

where g is the gene contain isoform iso.

Hence, we can control the data quality by rt, τ, and γ. In this study, we created two datasets by setting $rt = 10{,}000$, $\tau = 0.5$, $\gamma = 0.7$ and $rt = 12{,}500$, $\tau = 0.55$, $\gamma = 0.8$, which represent good and bad data qualities, respectively (Supplementary Fig. 36b). With expression of isoforms, we can produce the simulation dataset with Spanki simulator, and 1000 cells for each cell line were created.

Cell constitution of nPSC, hEE and iPSC datasets

nPSC contains 225 cells from 6 cell types, including early trophectoderm (Early-TE, *N*=18), epiblast (EPI, N=31), inner cell mass (ICM, *N*=22), inner cell mass-trophectoderm transition (ICM-TE, *N*=23), primitive endoderm (PE, *N*=14), and Trophectoderm (TE, *N*=117). The hEE dataset contains 90 cells from 7 cell types, including oocyte (*N*=3), zygote (*N*=3), 2-cell (*N*=6), 4-cell (*N*=12), 8-cell (*N*=20), morulae (*N*=16), late-blastocyst (*N*=30). The iPSC dataset contains 174 cells from 3 cell types, including induced progenitor stem cell (iPSC, *N*=63), motor neuron (MN, *N*=70), and neuron progenitor cell (NPC, *N*=41).

Processing of paired long-read and short-read scRNA-seq data

For long-read data processing, SiCeLoRe[5] (v2.1) was employed for cell barcode and UMI assignment, followed by genome mapping using minimap2[6] (v2.17) against the reference genome. Short-read data were processed using the 10x Genomics Cell Ranger[7] (v9.0.1) for genome alignment. Only cells with matched barcodes between long-read and short-read datasets were retained for downstream evaluation. UMI deduplication was performed using an in-house Python script. To preserve the maximum number of splicing-associated reads, junction reads, which were identified by the 'N' tag in the BAM CIGAR column, were prioritized during the deduplication process.

Supplementary Note 3. Execution of comparable algorithms

rMATS, BRIE1, BRIE2 (Aggregated Mode), Expedition, Psix, SCASL, and SCSES were executed on each of the datasets and generated splicing profiles. The count of neighbor cells in Psix was set to be 5 in all evaluation. In real datasets, other parameters in these methods were default setting. In artificial synthetic dataset, the parameters involving event filtering were set to remain events as many as possible in all methods. When evaluating the PSI accuracy, given that the SCASL quantifies splicing levels by AS probability, which represents the junction ratios in a junction module consisting of all junctions sharing the same 3'/5' splicing site, rather than the ratios of inclusion junction read counts to total junction read counts, we performed SCASL by only modifying the imputation targets from AS probabilities to junction read counts. Then the PSI values of SCASL were calculated based on the splicing events identified by SCSES. When evaluating cell clustering and pseudotime inferring, SCASL was performed without any modification.

Supplementary Note 4. Analysis and validation on Multiple myeloma

MM34 data analysis.

The analysis process was conducted with Seurat package in R. For gene expression data, the read counts were normalized and scaled in standard Seurat pipeline, while for splicing data, the normalization was omitted. Then, top 5000 highly variable features were selected for PCA analysis, respectively. The PCs, whose cumulative explained variance larger than 90%, were used to for find neighbor cells and UMAP projection. Leiden algorithm was used to find cell clusters with resolution=1. The marker genes of each cluster were defined as the genes, whose $\log_2 FC > 0.5$, $p < 0.05$ (Wilcoxon test, two-sided) and expressed fraction in either of two populations larger than 0.5. The pseudotime analysis as conducted with monocle3[8] and CytoTRACE[9] packages in R under default parameters. Top 10% of highly variable events were defined as highly variable events. The CNV states of MM cells were inferred with inferCNV R package, where the expression data of normal BM plasm cells and B cells, downloaded from Tabula Sapiens[10], were used as reference. CNV scores were defined as the sum of the squares

of all segment abnormal copy numbers[11]. SCSES modified RNA velocity replaced the genes with RI events. The junction read counts and retained read counts of RI events were imputed by cell similarities, and then were used as spliced and unspliced read counts, respectively. The RNA velocity was conducted with scVelo[12] package in Python. The DSEs were defined as the events, whose $\Delta PSI > 0.1$ and $FDR < 0.05$. The events without gene differentially expressing satisfied the property that the associated genes are expressed in more than 80% cells, additionally.

Validation of Bortezomib (BTZ)-resistance cell subgroups in independent multiple myeloma (MM) dataset

To independently validate our findings in patient MM34, we analyzed an additional scRNA-seq data (GSE118900)[13], which was collected from a primary MM patient (NDMM3). Based on imputed splicing profiles by SCSES, we identified two cell subgroups (SC1 and SC2) that were not detectable by conventional gene expression analysis (Supplementary Fig. 22a). Then, we identified the differentially spliced events (DSEs) between SC1 and SC2 (ΔPSI>0.1, FDR<0.05) and found that 64 events were differentially spliced in both MM34 and NDMM3 samples (Supplementary Fig. 22b), 44 of which had a consistent splicing direction (*P*-value=0.035, Fisher's exact test). Interestingly, by using the splicing intensities of 44 DSEs, most SC1 and SC2 cells from the two patients were clustered together. More intriguingly, the partial of SC2 cells, but no SC1 cells from two patients were clustered together with SC3 and SC4 cells from the relapsed sample (Supplementary Fig. 22c). This observation supports our hypothesis in the initial submission that SC2 cells had BTZ-resistance potential at primary diagnosis. Additionally, the activity of MM34-SC2 marker genes estimated by UCell[14] was significantly higher in NDMM3-SC2 cells than in NDMM3-SC1 cells (Supplementary Fig. 22d). These results confirmed that we have successfully recapitulated the existence of a novel cluster SC2 in an independent scRNA-seq data from a primary MM patient. This novel cluster in the primary tumor is potentially associated with tumor relapses upon the BTZ treatment, and could only be detected by SCSE rather than gene expression clustering.

To assess any clinical relevance of SC2 cells, we analyzed three independent bulk RNA-seq datasets (MMRF[15]: *N*=566, GSE9782[16]: *N*=188, and GSE24080[17]: *N*=559) of BTZ-treated

cohorts. The activity of MM34-SC2 marker genes was calculated via UCell[14] or GSVA[18] for each patient, and patients were separated into two equal-sized groups based on the median value of SC2 marker genes' activity. The results showed that the patients with high activities had significantly shorter overall survival time (Fig. 4k) and shorter disease-free time (Fig. 4l) (log-rank test, *P*-value < 0.0001). This implies that SC2 maker genes were associated with patient outcome. Furthermore, primary diagnosis samples from eventually relapsed patients exhibited higher SC2 marker activity and lower SC1 activity compared to non-relapsed patients, suggesting SC2-like cells are associated with higher risk of BTZ resistance and tumor relapse (Supplementary Fig. 22e).

Supplementary Note 5. Analysis on Embryo stem cell

Embryo stem cell data analysis

The analysis process was conducted with Seurat package in R. Top 40% highly variable features was selected for PCA, and PCs retained 70% of the variation in the data was kept for UMAP projection and detection of nearest neighbors. Top 30% highly variable genes and splicing events were used to cluster the cells at 36h. Leiden algorithm was used to identify clusters with a resolution of 0.5. The marker splicing events of each cluster were defined as events with $\Delta PSI > 0.1$ and $FDR < 0.05$ (Wilcoxon test, two-sided), as well as mean TPM of the target gene in either group was greater than 1. The marker genes of each cluster were defined as genes whose $\log_2 FC > 0.25$, $P < 0.05$ (Wilcoxon test, two-sided), and the gene expression fraction in at least one of the two clusters was greater than 0.5. The RNA velocity was conducted with scVelo. The regulatory network was constructed for differentially expressed RBPs and differentially spliced events. The relationships with $|SCC| > 0.4$ and $FDR < 0.05$ were used to construct the network. The regulatory network was visualized using Cytoscape software (v3.10.1). Genes with $\log_2 FC > 0.1$ between any two adjacent time points were selected to measure the differentiation degree. Gene signature scores were calculated with the AddModuleScore_UCell function from the UCell package (v2.2.0) using default parameters[19]. The differentiation order of each cell was determined by the gene signature scores.

Correlation analysis between 72h- and 96h-cells in embryo stem cell data

Most cells at different time points were clustered into independent groups by SCSES splicing profile, indicating clear splicing dynamics throughout the differentiation course (Fig. 5a), while a small set of cells from 96h were overlapped with cells from 72h (Fig. 5a). As described in the previous study where the dataset was generated[20191817], the authors stated that with a general decrease of differentially expressed genes from 0h to 96h, “cells could gradually transition into a relatively ‘stable’ state at 72h of differentiation”. To validate this statement, we compared the expression levels of *CXCR4* and *SOX17*, both of which are definitive endoderm marker genes[21201918], and found no significant changes between 72h and 96h (Supplementary Fig. 23a, b). Additionally, we also found that the number of differentially spliced events also gradually decreased after 72h (Supplementary Fig. 23c). These findings support the authors' conclusion that cells reach a relatively stable differentiation state by 72h.

To further investigate the heterogeneity among 96h-cells, we stratified the 96h-cells into two subpopulations, based on their distances to 72h-cells (Supplementary Fig. 23d, e). The 96h-cells overlapping with 72h-cells were labeled as “96-overlap”, and others were labeled as “96h-specific”. CytoTrace-based analysis of cellular stemness revealed that the 96-overlap cells showed greater developmental potential (Supplementary Fig. 23f), implying an incomplete differentiation state. Furthermore, we detected 253 differentially spliced events between 96h-cells and other cells, and 245 of which were detected with the same alteration direction between 96-specific cells and 96-overlapped cells (Supplementary Fig. 23g). All these results indicate that 96-specific cells were in a more mature stage compared to 96-overlap cells.

Supplementary Note 6. Analysis on mouse hematopoietic stem cell inDrop-seq data

Cell metadata were downloaded from https://github.com/AllonKleinLab/paper-data/tree/master/Lineage_tracing_on_transcriptional_landscapes_links_state_to_fate_during_differentiation. The FASTQ files were demultiplexed using the inDrops pipeline. LARRY pipeline (https://github.com/AllonKleinLab/LARRY) was used to call lineage barcodes. Cells with library size less than 1,000, the number of detected genes less than 500, or mapping rate to

mitochondrial genes greater than 20% were removed. The remaining dataset contained 30,686 cells. featureCounts was employed to assign gene names to each reads of the alignment files. UMI-tools[22212019] was used to quantify the number of reads per gene based on the gene names and the UMI attached to the read. To adapt these ALE events to the SCSES framework, we extracted the ALE sequence features based on the structure of ALE (Supplementary Fig. 38, Supplementary Data 14). Without changing other procedures, we estimated the splicing levels of these ALE events. Top 1500 highly variable features were selected for gene expression profile and splicing profile, respectively. A total of 3,000 combined highly variable features were selected for PCA, and PCs retaining 70% of the variation in the data were kept for UMAP projection and finding nearest neighbors. The Leiden algorithm was used for cell clustering, with a resolution parameter set to 0.5. For the cells of monocyte subgroups, top 1,500 highly variable genes and AS events were selected for PCA, respectively. PCs retaining 70% of the variation were applied Leiden algorithm for further clustering (resolution = 0.2). The marker splicing events of each monocyte subgroups were defined as events whose $\Delta PSI > 0.1$ and $FDR < 0.05$ (Wilcoxon test, two-sided) and the mean normalized UMI counts of the target gene in either group were greater than 0.3. The marker genes of each cluster were defined as genes whose $\log_2 FC > 0.25$, $FDR < 0.05$ (Wilcoxon test, two-sided) and the gene expression fraction in at least one of the clusters was greater than 0.3. The autophagy-related genes were derived from the GO database. To identify the late-stage cells derived from each monocyte subgroup, we selected specific clone ids in each subgroup based on clonal information in the dataset. Subsequently, cells on day 6 with the subgroup-specific clone ids were defined as late-stage cells corresponding to the subgroup.

Supplementary Note 7. Parameter selection of SCSES

Recommendation for cellular similarity matrix selection

Benefiting from the higher coverage in RBP genes than junctions, the cellular similarities based on RBP expression are fitted in most cases. However, RBP expressions cannot comprehensively represent cellular splicing profiles. RNA splicing is a complex process, where RBP expressions are only one of several regulatory factors[23]. As a result, junction read counts and PSI values depict cell splicing states more directly. Comparing between

junction read counts and PSI values, PSI values are the downstream product of junction read counts and may be affected by the quality of junction read counts.

Regarding the strategy for feature selection, the evaluation in the manuscript indicate that the performances of the three similarity measures are highly dataset-dependent. We recommend that users test and compare the results obtained from different similarity measures to determine the most suitable approach for their specific dataset. Based on the above analysis of advantages and disadvantages, we suggest a prioritized ranking of the measures as follows: RBP expression, junction read counts, and PSI. This recommendation is particularly valuable for datasets with lower coverage, where the expression of RBP genes may provide more robust and reliable insights into splicing regulation.

The rationale for the strategy choices

In SCSES, we use three imputation strategies for four types of biological scenarios of event-cell pairs. The strategies choices for different scenarios were determined based on our experimental results on cell line datasets. As shown in Supplementary Fig. 7, compared to other strategies, PSI values estimated by Strategy 1 in single-cell dataset showed higher Spearman correlation coefficients (SCC) with reference PSI values derived from matched bulk data in ND (non-dropout) group, which suggests that Strategy 1 is more suitable for ND group pairs. In the WD (with-dropout) group, we evaluated the percentage of true-positive event-cell pairs (where ΔPSI between reference and imputed PSI was <0.1), because the real PSI values for BD group are either zero or one. The results demonstrated Strategy 2's superior performance for both BD (biological dropout) and TD (technical dropout) groups (Supplementary Fig. 7). Furthermore, we observed that some event-cell pairs could not obtain information from neighbor cells when those neighbors also belonged to the TD group. We therefore tested Strategy 3's performance separately in TD+Info and TD-Info groups. The results showed Strategy 2 achieved minimal RMSE in TD+Info cases, while Strategy 3 performed better in TD-Info cases. Hence, Strategy 3 is used to PSI imputation in TD-Info group pairs. To validate the robustness of these scenario-dependent patterns, we replicated the analysis in down-sampled datasets of iPSC, hEE and nPSC, using raw PSI values as reference. Similar patterns can be found in these datasets

(Supplementary Fig. 8).

Biologically, this observation makes sense: ND events have detectable reads supporting both inclusion and exclusion junctions, thereby ensuring the inherent reliability of their PSI values. In contrast, WD events suffer from uncertainty about whether missing reads reflect true biological absence or technical limitations, making junction read imputation (Strategy 2) a more robust approach. Event similarity (Strategy 3) provides supplementary information only when neighboring cells are unable to provide sufficient read coverage for reliable inference.

Analysis of hyperparameters

In SCSES framework, some hyperparameters should be pre-determined, including restart probability (alpha), convergence thresholds (decay) of random walk, and K value for the diffused event similarity matrix. We evaluated the sensitivity of SCSES to key hyperparameters through a series of experiments. To evaluate the accuracy of PSI estimation, we calculated the Spearman correlation coefficients (SCC) between the PSI values estimated by SCSES and the benchmark PSI values (derived from bulk RNA-seq of the matched cell type) across all events within each cell in four single-cell datasets of cell lines. To evaluate the accuracy of cell clustering, we calculated the normalized mutual information (NMI) between the K-means clustering results derived from the estimated PSI profiles and the cell type annotations provided in the original publications of the test datasets across down-sampled datasets of iPSC, hEE and nPSC.

For the restart probability (λ), we tested six candidates (0, 0.1, 0.2, 0.4, 0.6, 0.8). As shown in Supplementary Fig. 32, the PSI estimation accuracy remained stable when λ was larger than 0.1, while the clustering accuracy achieved better results when λ was set to 0.2. After comprehensive evaluation across multiple datasets, we set λ =0.2 as our default parameter.

For the K value for the diffused similarity matrix, the K values of cell similarities are automatically determined by the dynamic K algorithm for each cell. We further tested the effect of different K values of event similarities by evaluating six candidates (5, 10, 15, 20, 30, 50). As shown in Supplementary Fig. 31, the K values of event similarities had no obvious influence on the PSI estimation accuracy, while the clustering accuracies exhibited moderate fluctuations. After comprehensive evaluation across multiple datasets, we set K=10 as our default parameter.

For the convergence thresholds (decay) of random walk, we tested seven candidates (Inf, 0.5, 0.1, 0.05, 0.01, 0.001, 0.0001), where Inf means no diffusion procedure was applied. As shown in Supplementary Fig. 33, the PSI estimation accuracy increased as the decay decreased, and became relatively stable after 0.1. The clustering accuracies showed no consistent patterns across the three datasets. After comprehensive evaluation across multiple datasets, we set decay=0.05 as our default parameter.

It is worth noting that all these hyperparameters are customizable in the SCSES package. Users can test with different hyperparameter combinations to select the most suitable ones according to their specific situation.

Ablation studies

We conducted ablation studies to evaluate the contribution of imputation strategy integration, similarity diffusion and fine-tune procedure of the scenario decision model of event-cell pairs

Firstly, we compared the performance of the integrated strategy with that of individual strategies, which included: Strategy 1 (imputing raw PSI using cell similarity), Strategy 2 (imputing raw junction read counts using cell similarity), and Strategy 3 (imputing the results from Strategy 2 using event similarity). We demonstrated that the integrated approach outperformed the individual strategies in most cases, particularly in terms of PSI accuracy and cell clustering (Supplementary Fig. 28). For example, in the HCT116 dataset, the integrated strategies showed higher SCC values between the predicted PSI values and benchmark PSI values compared to the individual strategies, indicating more accurate PSI estimation. Additionally, in the nPSC dataset, the NMI values for cell clustering using the integrated strategy were consistently higher than those from the individual strategies, suggesting more consistent clustering with the real cell types.

Secondly, we evaluated the contribution of similarity diffusion to the overall performance of the algorithm. As shown in Supplementary Fig. 29, the diffusion procedure improved the accuracy of both PSI estimation and cell clustering in most cases. For instance, in the HCT116 dataset, applying diffusion increased the SCC values between predicted and benchmark PSI values by at least 0.05. In the iPSC dataset, the diffusion step led to a 70%

improvement in NMI for cell clustering. These results highlight the importance of similarity diffusion in enhancing the final splicing profile.

Finally, we evaluated the contribution of the fine-tuning procedure in the scenario decision model. The pre-trained model was originally trained on single-cell cell line datasets. To assess the benefit of fine-tuning, we used down-sampled datasets from iPSC, hEE, and nPSC. As shown in Supplementary Fig. 30, the fine-tuned model consistently outperformed the pre-trained model across all cases. For example, in the iPSC dataset, fine-tuning increased the SCC between predicted and benchmark PSI values by more than 0.1, and improved the NMI for cell clustering by over 30%. These results demonstrate that the fine-tuning procedure enables the model to better adapt to specific datasets.

Supplementary Figures

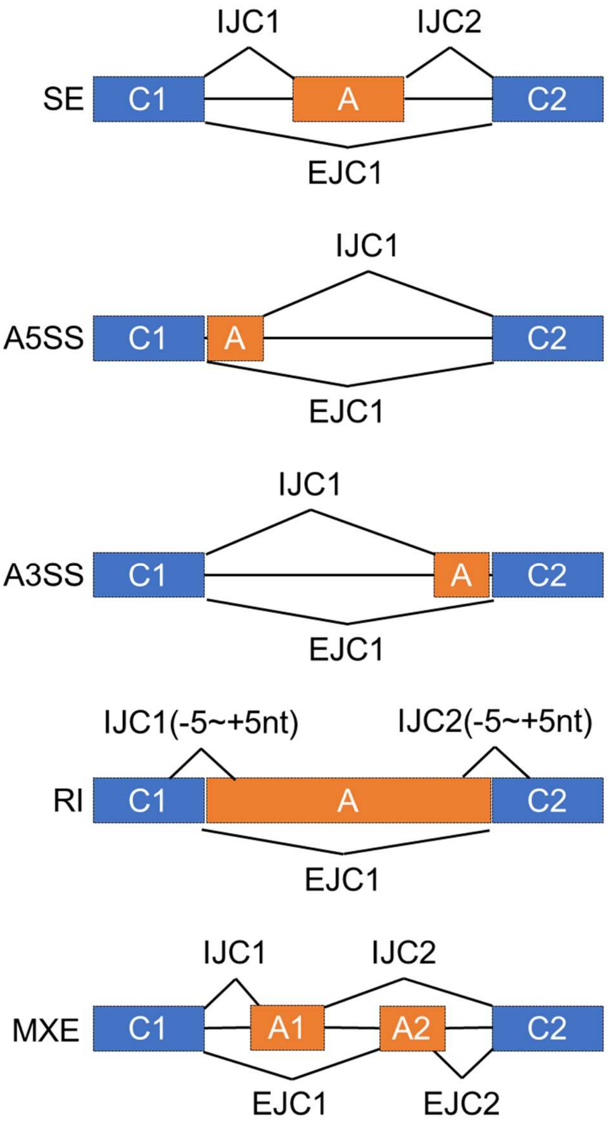

$$PSI_{SE} = \frac{(IJC1 + IJC2)/2}{EJC1 + (IJC1 + IJC2)/2}$$

$$PSI_{A5SS} = \frac{IJC1}{IJC1 + EJC1}$$

$$PSI_{A3SS} = \frac{IJC1}{IJC1 + EJC1}$$

$$PSI_{RI} = \frac{(IJC1 + IJC2)/2}{EJC1 + (IJC1 + IJC2)/2}$$

$$PSI_{MXE} = \frac{(IJC1 + IJC2)/2}{(EJC1 + EJC2)/2 + (IJC1 + IJC2)/2}$$

Supplementary Figure 1: The definition of PSI of different AS events. IJC1/IJC2 represents the inclusion junction read counts, while the EJC1/EJC2 represents the exclusion junction read counts. For RI events, the IJC1 and IJC2 are defined as the reads spanning 5nt upstream to downstream of the cryptic 3'/5' splicing site, respectively. "A" represents the alternative splicing region. "C1/C2" represents the constitutive exon.

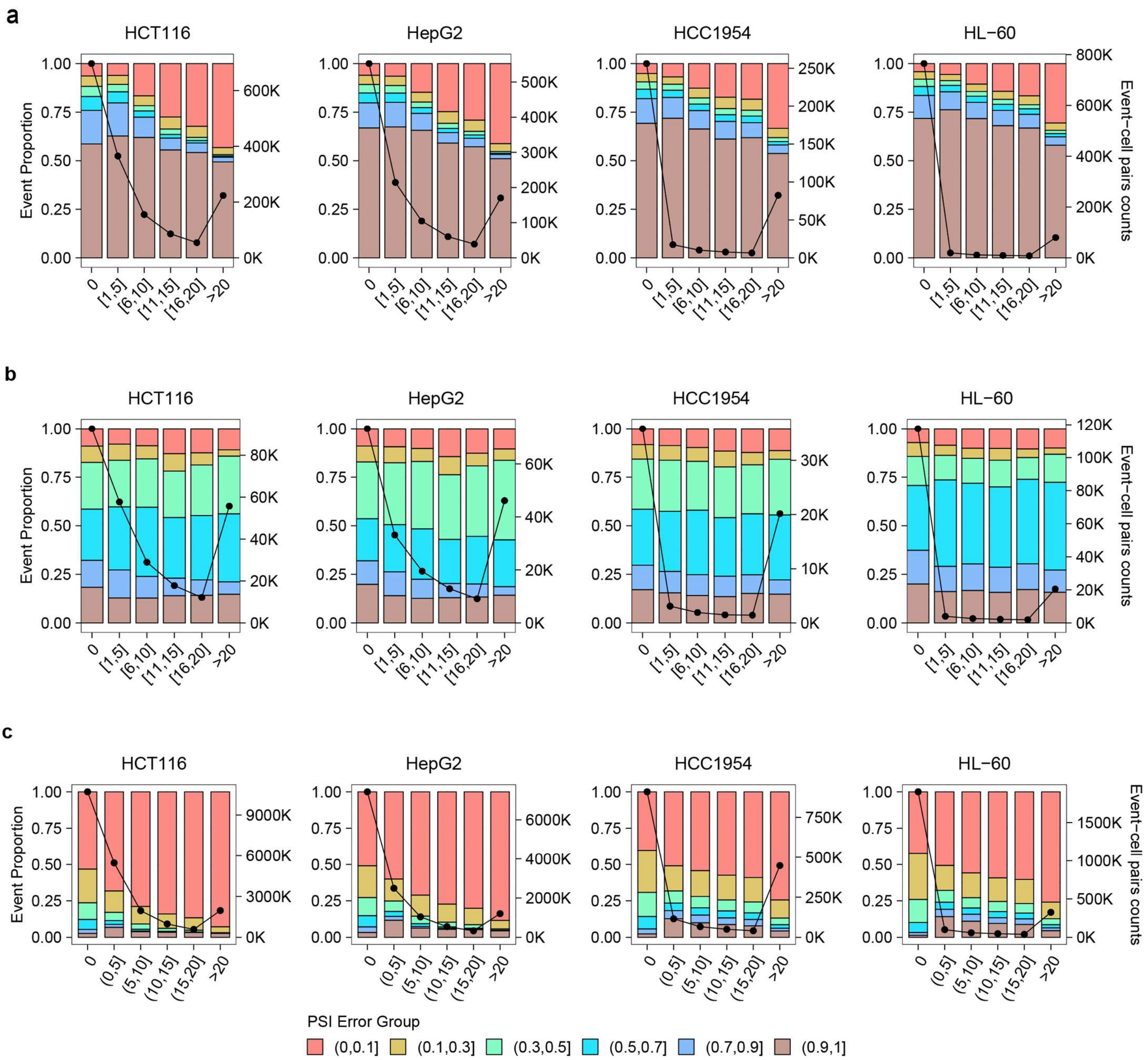

Supplementary Figure 2: The relationship between junction read counts and PSI values accuracy in Expedition and SCASL. Bar plots showing the distribution of absolute difference between the benchmark and the estimated splicing levels for Expedition (**a**, **b**) and SCASL (**c**) across four cell lines. The splicing levels in matched bulk data were calculated as benchmark splicing levels. Each event-cell pair was stratified into six groups based on total junction read counts (x-axis), and the PSI differences were binned into six intervals ranging from 0 to 1 (y-axis). Points within each bar indicate the count of event-cell pairs. (**a**) SE events analyzed by Expedition; (**b**) MXE events analyzed by Expedition; (**c**) All events analyzed by SCASL.

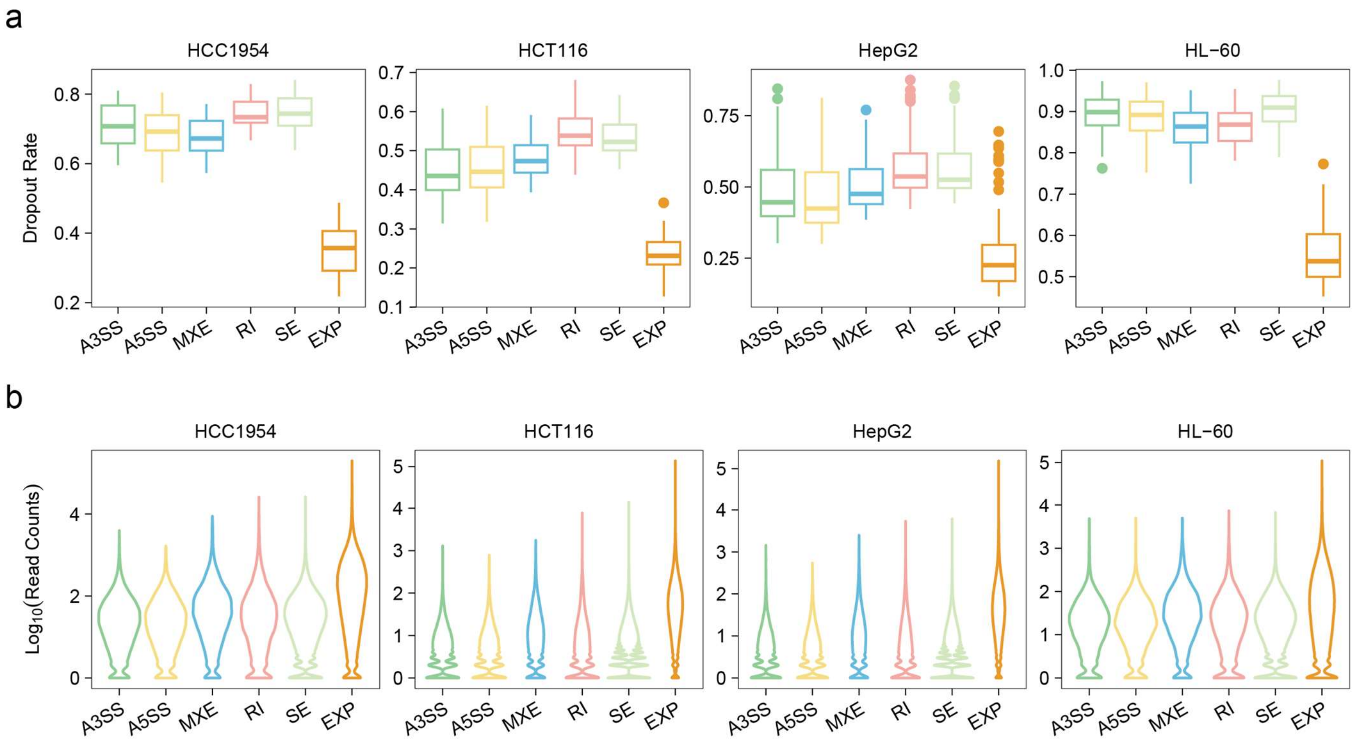

Supplementary Figure 3: Analysis of splicing event sparsity. (**a**) Box plots showing the distribution of dropout rate per cell across different alternative splicing event types (column 1-5) and gene (column 6). N_{HCC1954} =22, N_{HCT116}=91, N_{HepG2} =68, $N_{\mathrm{HL\text{-}60}}$=54. The dropout rate in each cell is defined as the proportion of features (splicing events or genes) without any supporting read. The distribution of dropout gene proportions is shown as a reference. The boxes indicate median (center), Q25, and Q75 (bounds of box), the smallest value within 1.5 times interquartile range below Q25 and the largest value within 1.5 times interquartile range above Q75 (whiskers). (**b**) Violin plots showing the distribution of total read counts per cell across different alternative splicing event types (column 1-5) and gene (column 6), only considering non-dropout features (splicing events or genes). The distribution of gene expression read counts is shown as a reference. N_{HCC1954} =22, N_{HCT116}=91, N_{HepG2} =68, $N_{\mathrm{HL\text{-}60}}$=54.

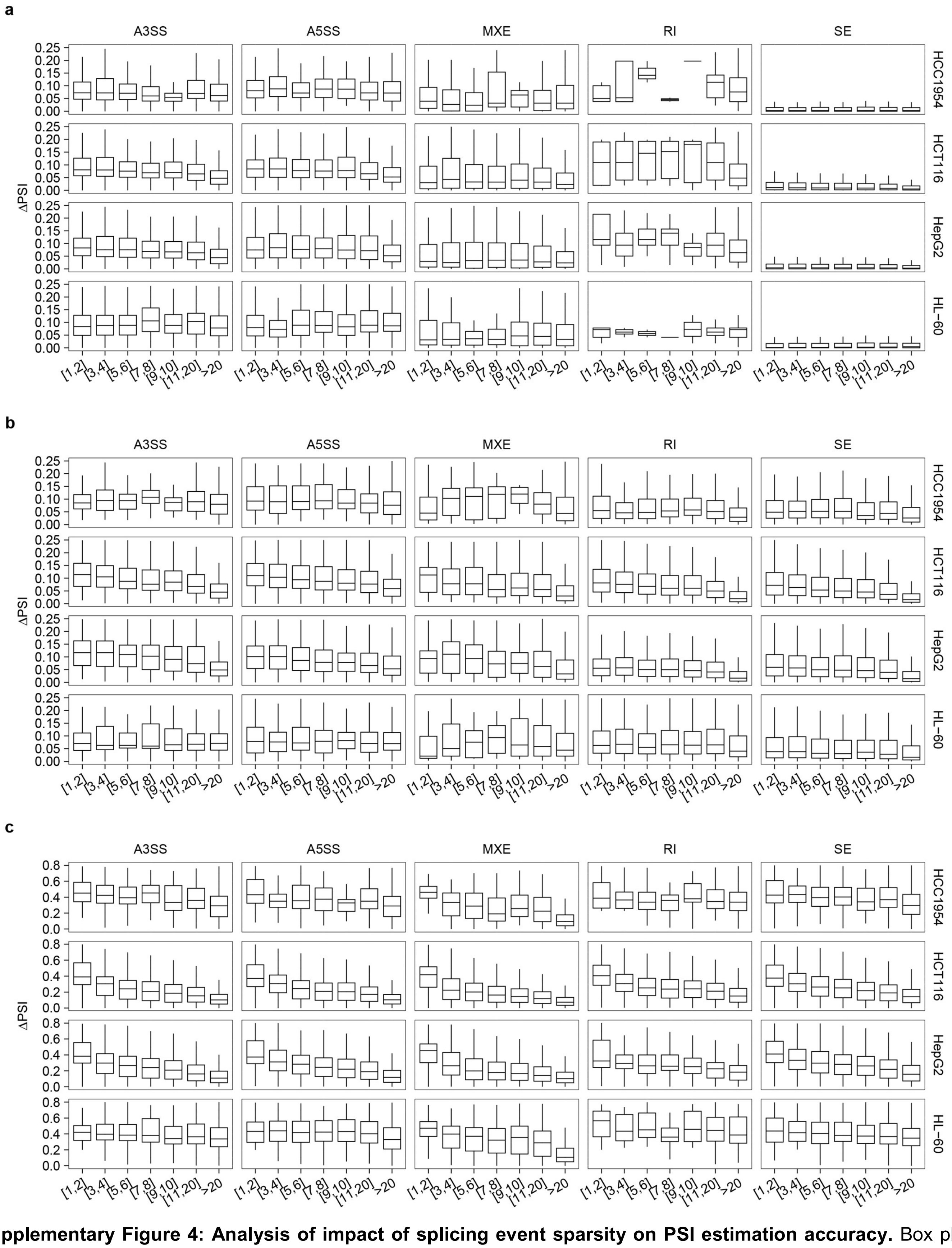

Supplementary Figure 4: Analysis of impact of splicing event sparsity on PSI estimation accuracy. Box plots showing the distribution of PSI differences between raw single-cell data and corresponding benchmarks for each splicing event, grouped by total read counts. The boxes indicate median (center), Q25, and Q75 (bounds of box), the smallest value within 1.5 times interquartile range below Q25 and the largest value within 1.5 times interquartile range above Q75 (whiskers). (**a**) High-PSI event group (defined as events with PSI > 0.8 in all four cell lines); (**b**) Low-PSI event group (defined as events with PSI < 0.2 in all four cell lines); (**c**) Normal-PSI event group (defined as events that are not classified in either the High or Low Group).

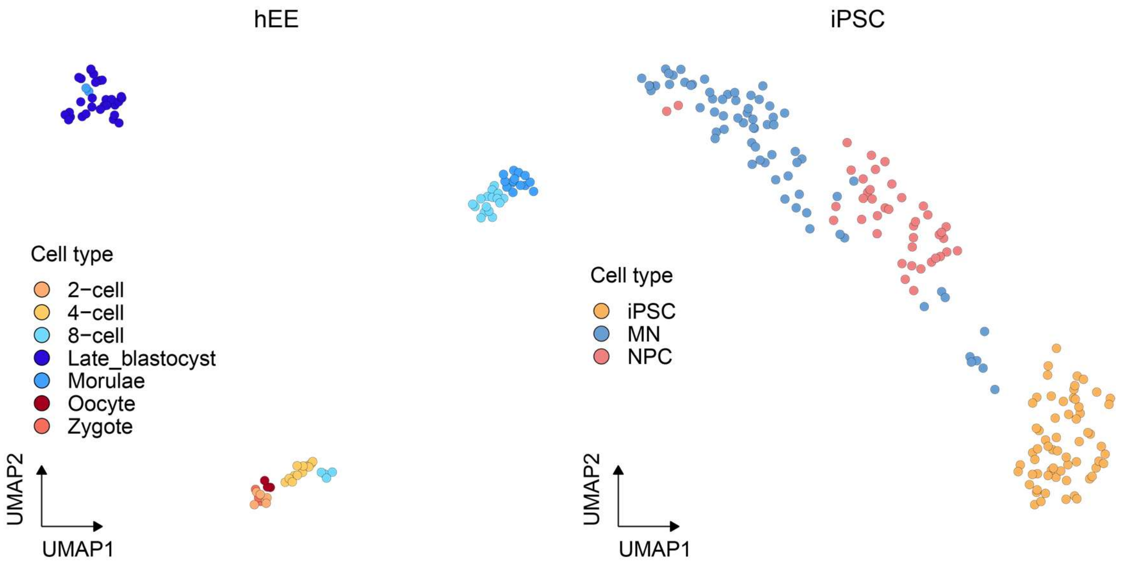

Supplementary Figure 5: The validation of the assumption: cells with similar splicing machinery activity display similar splicing patterns. UMAP plots based on the original AS profiles showing specific cell types during human embryo stem cell differentiations (**left panel**) and differentiation states of induced human pluripotent stem cells (**right panel**). N_{Oocyte}=3, N_{Zygote}=3, $N_{\text{2-cell}}$=6, $N_{\text{4-cell}}$=12, $N_{\text{8-cell}}$=20, N_{Morulae}=16, $N_{\text{Blastocyst}}$=30. N_{iPSC}=63, N_{MN}=70, N_{NPC}=41.

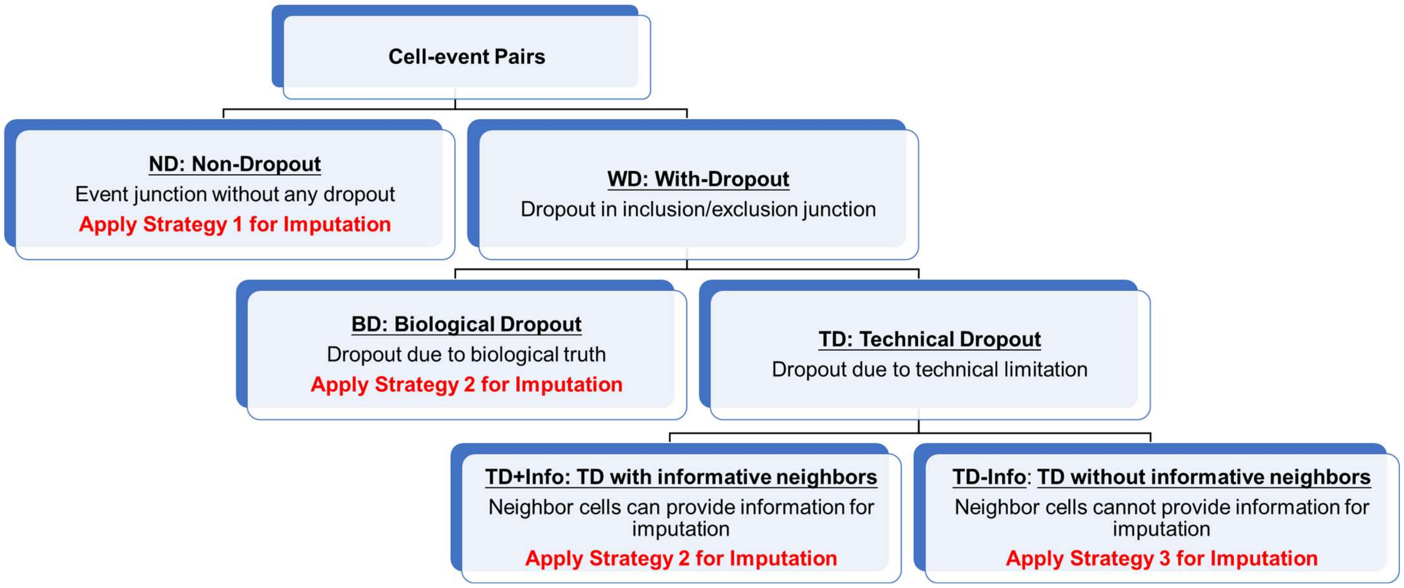

Supplementary Figure 6: The hierarchical structure of four categories of cell-event pairs. Event-cell pairs with dropout in either inclusion or exclusion junctions are defined as WD (with dropout), while others are defined as ND (non dropout). In WD, event-cell pairs are categorized into BD for biological dropout or TD for technical dropout. In BD, both target and neighbor cells harbor good reads depth but lacking alternative junction reads, which imply a fixed isoform for this event. In contrast, the target cell of TD gets both low reads depth and limited alternative junction reads. Based on the abundance of alternative reads in neighbor cells, TD could be further classified into TD+Info that knowledge could be learned from local neighbors with rich splicing information, while others as TD-Info.

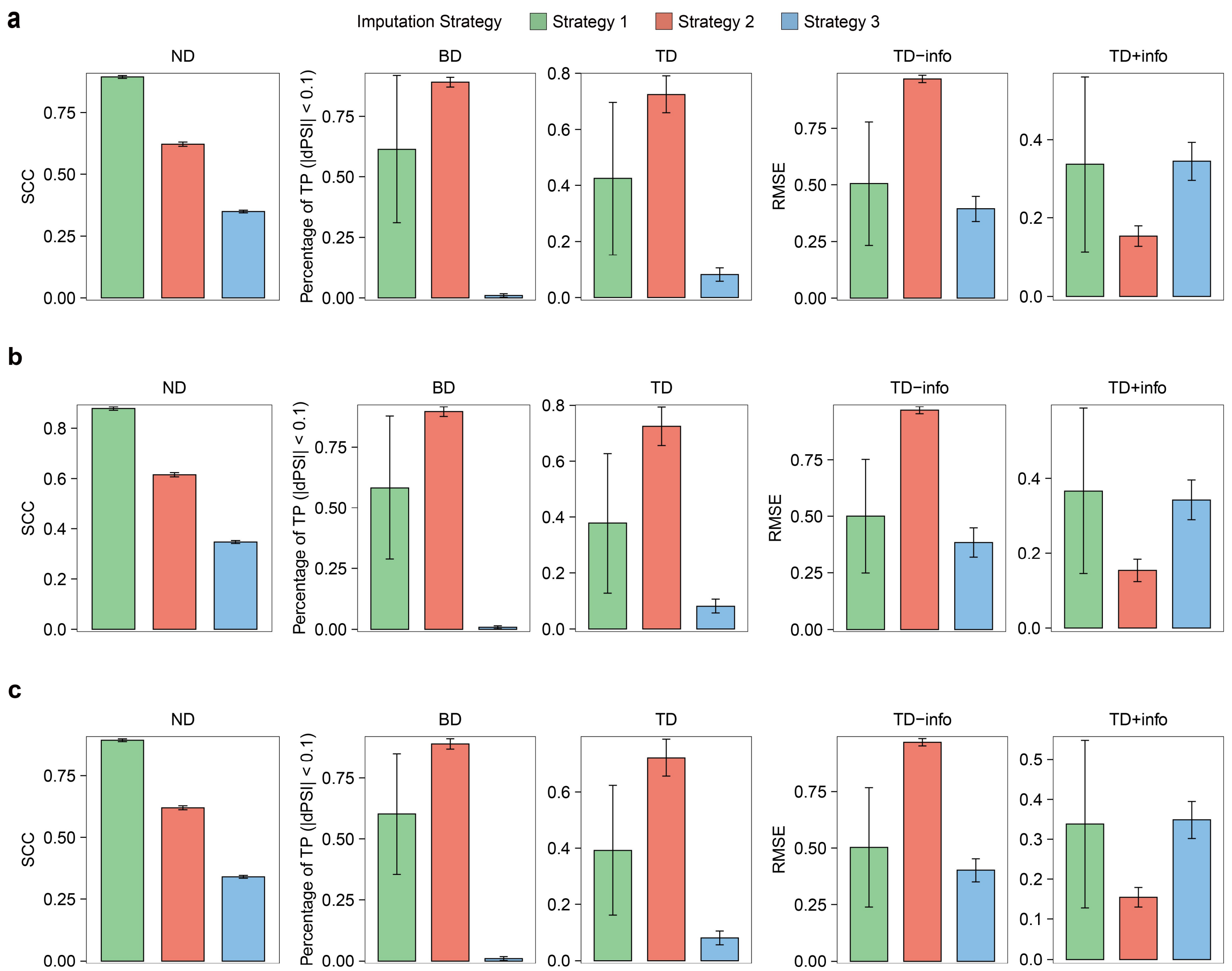

Supplementary Figure 7: Performance of different imputation strategies for different event-pair scenarios with cell similarities computed by RBP (a), PSI (b), RC (c) in cell line dataset. SCC represents the SCC between imputed PSI and benchmark PSI in cells. TP means the event-cell pairs, whose absolute differences between imputed PSI and benchmark PSI is less 0.1. The TP percentage indicates the proportion of TP pairs among BD or TD pairs. RMSE shows the root mean square error between imputed PSI and benchmark PSI in cells. Colors represent different imputation strategies. ND: non-dropout group, BD: biological dropout group, TD: technical dropout group, TD+info: technical dropout group that could obtain information from neighbor cells, TD-info: technical dropout group that could not obtain information from neighbor cells. Error bars represent the standard error of the mean in all event-cell pairs.

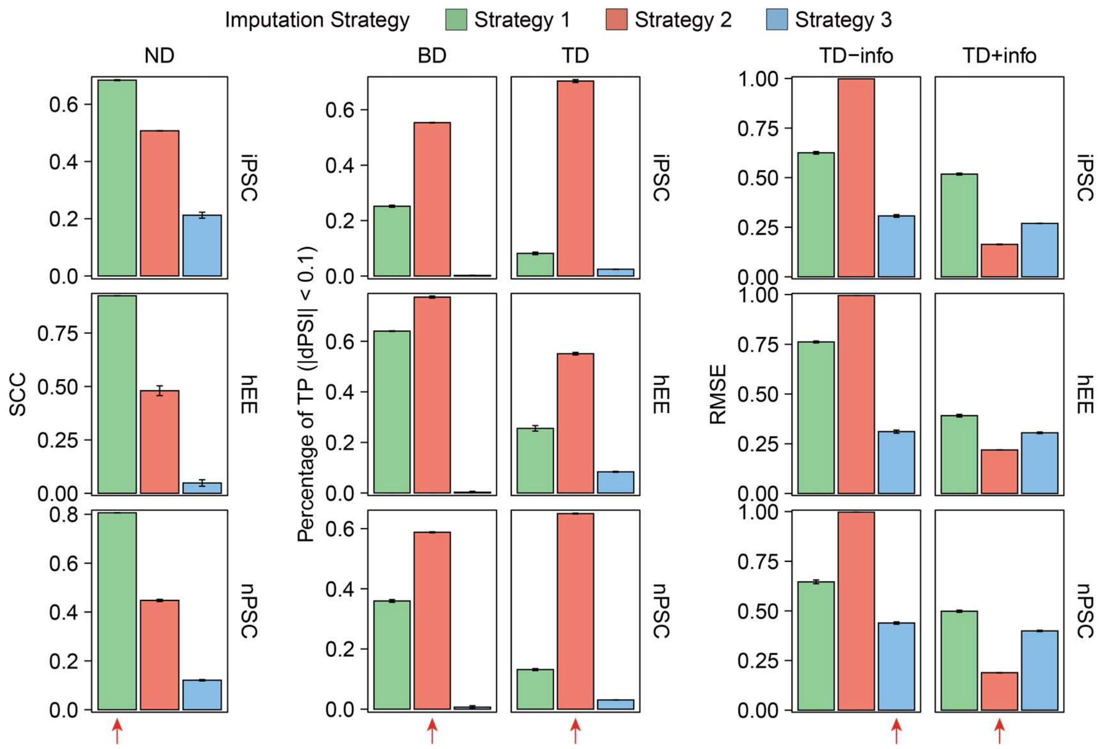

Supplementary Figure 8: Performance of different imputation strategies for different event-pair scenarios with cell similarities computed by RBP in iPSC, hEE, and nPSC down-sampling datasets. SCC represents the SCC between imputed PSI and benchmark PSI in cells. TP means the event-cell pairs, whose absolute differences between imputed PSI and benchmark PSI is less 0.1. The TP percentage indicates the proportion of TP pairs among BD or TD pairs. RMSE shows the root mean square error between imputed PSI and benchmark PSI in cells. Colors represent different imputation strategies. The red arrow indicates the strategy used in SCSES. ND: non-dropout group, BD: biological dropout group, TD: technical dropout group, TD+info: technical dropout group that could obtain information from neighbor cells, TD-info: technical dropout group that could not obtain information from neighbor cells. Error bars represent the standard error of the mean in all event-cell pairs.

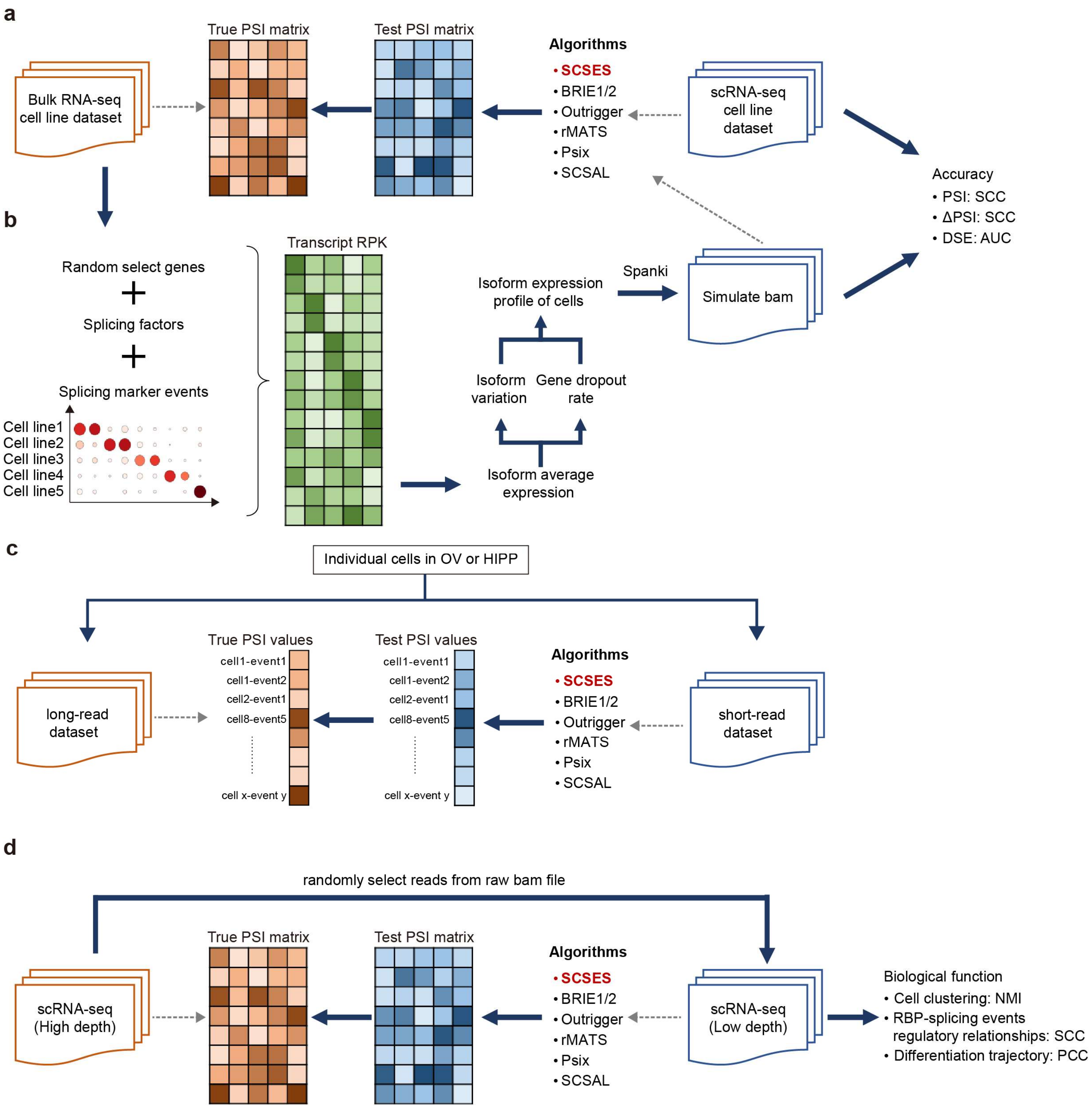

Supplementary Figure 9: Evaluation pipeline of SCSES

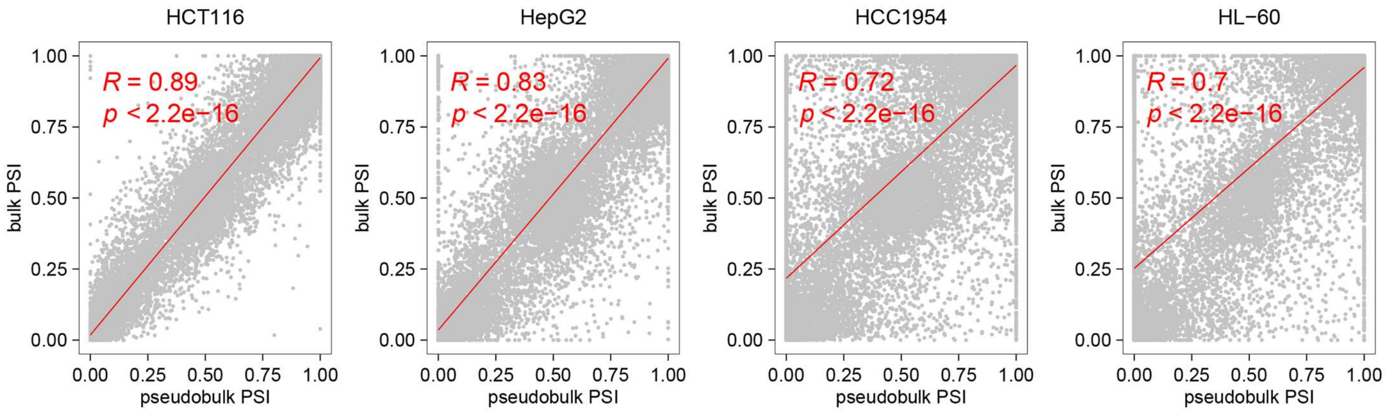

Supplementary Figure 10: PSI consistency comparison between bulk and pseudo-bulk data in four cell lines. Scatter plots show correlations with Spearman correlation coefficients (R) and P-values indicated.

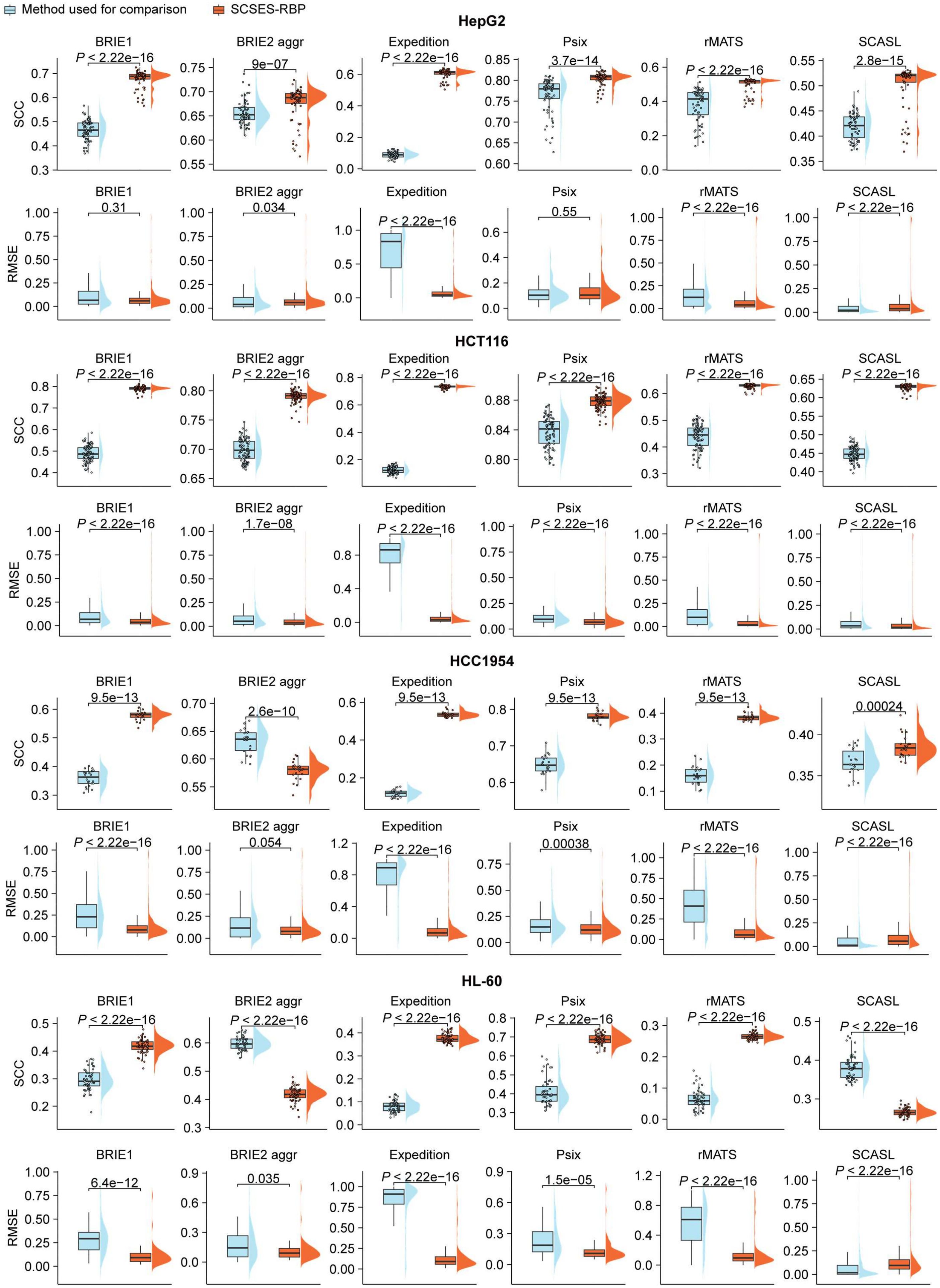

Supplementary Figure 11: Performance evaluation of SCSES-RBP against existing algorithms on cell line scRNA-seq data. Raincloud plots showing the performance comparison between SCSES-RBP and other algorithms in real scRNA-seq data of HepG2, HCT116, HCC1954, and HL-60. The events detected by both SCSES and the compared

algorithm are considered for each comparison. The SCC value refers to the correlation between estimated PSI values and benchmarks of all events in each cell. $N_{HCC1954}$ =22, N_{HCT116}=91, N_{HepG2} =68, N_{HL-60}=54. The RMSE is calculated between estimated PSI and benchmarks of an event among all cells. N_{BRIE1}=556, $N_{BRIE2\ aggr}$=556, $N_{Expedition}$=1,458, N_{Psix}=495, N_{rMATS}=10,313, N_{SCASL}=10,313. The boxes indicate median (center), Q25, and Q75 (bounds of box), the smallest value within 1.5 times interquartile range below Q25 and the largest value within 1.5 times interquartile range above Q75 (whiskers). The *P*-values are calculated by the Wilcoxon test (two-sided test). For pairwise comparisons, unadjusted *P*-values are reported.

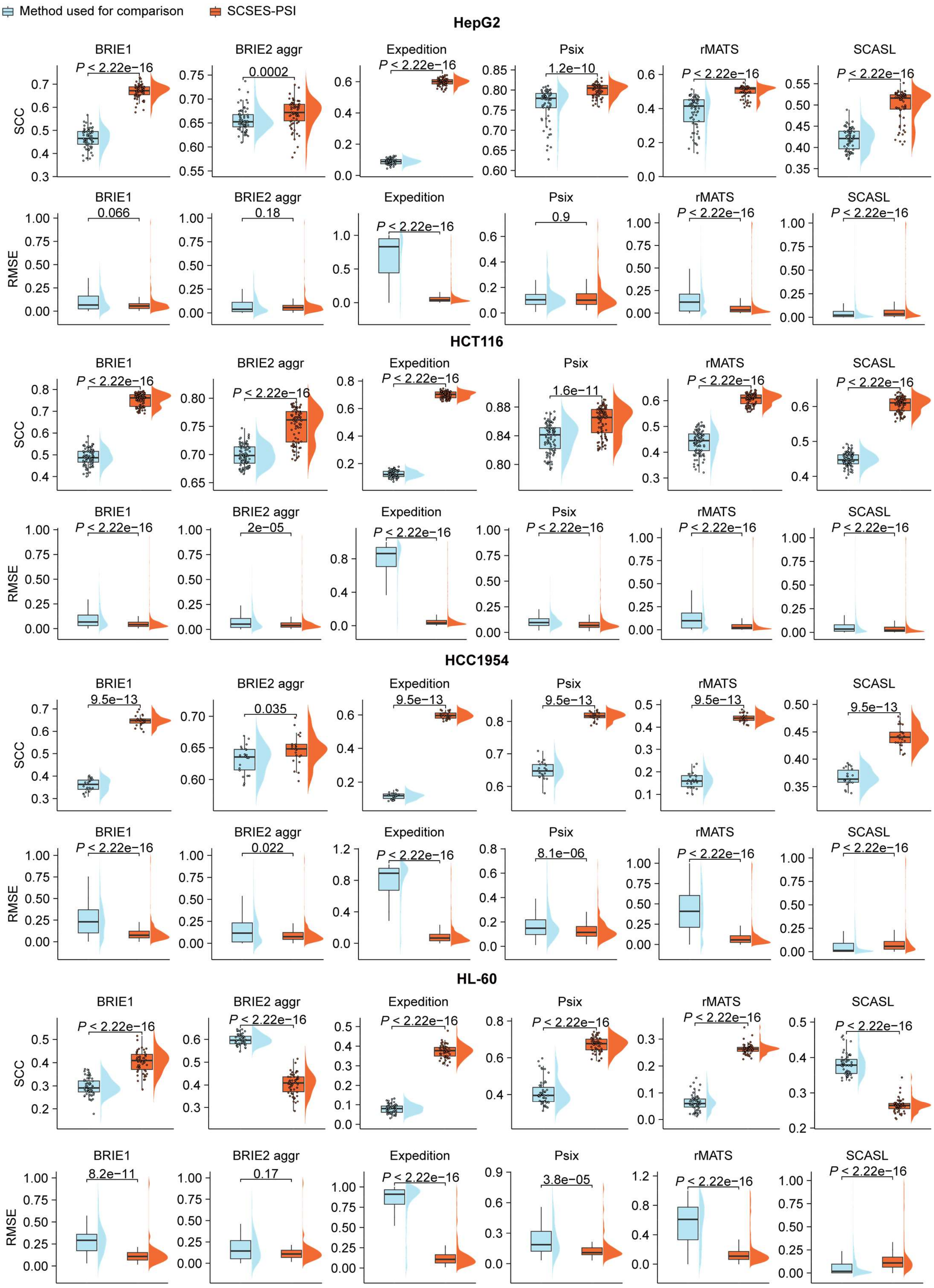

Supplementary Figure 12: Performance evaluation of SCSES-PSI against existing algorithms on cell line scRNA-seq data. Raincloud plots showing the performance comparison between SCSES-PSI and other algorithms in

real scRNA-seq data of HepG2, HCT116, HCC1954, and HL-60. The events detected by both SCSES and the compared algorithm are considered for each comparison. The SCC value refers to the correlation between estimated PSI values and benchmarks of all events in each cell. $N_{HCC1954}$ =22, N_{HCT116}=91, N_{HepG2} =68, $N_{HL\text{-}60}$=54. The RMSE is calculated between estimated PSI and benchmarks of an event among all cells. N_{BRIE1}=556, $N_{BRIE2\ aggr}$=556, $N_{Expedition}$=1,458, N_{Psix}=495, N_{rMATS}=10,313, N_{SCASL}=10,313. The boxes indicate median (center), Q25, and Q75 (bounds of box), the smallest value within 1.5 times interquartile range below Q25 and the largest value within 1.5 times interquartile range above Q75 (whiskers). The *P*-values are calculated by the Wilcoxon test (two-sided test). For pairwise comparisons, unadjusted *P*-values are reported.

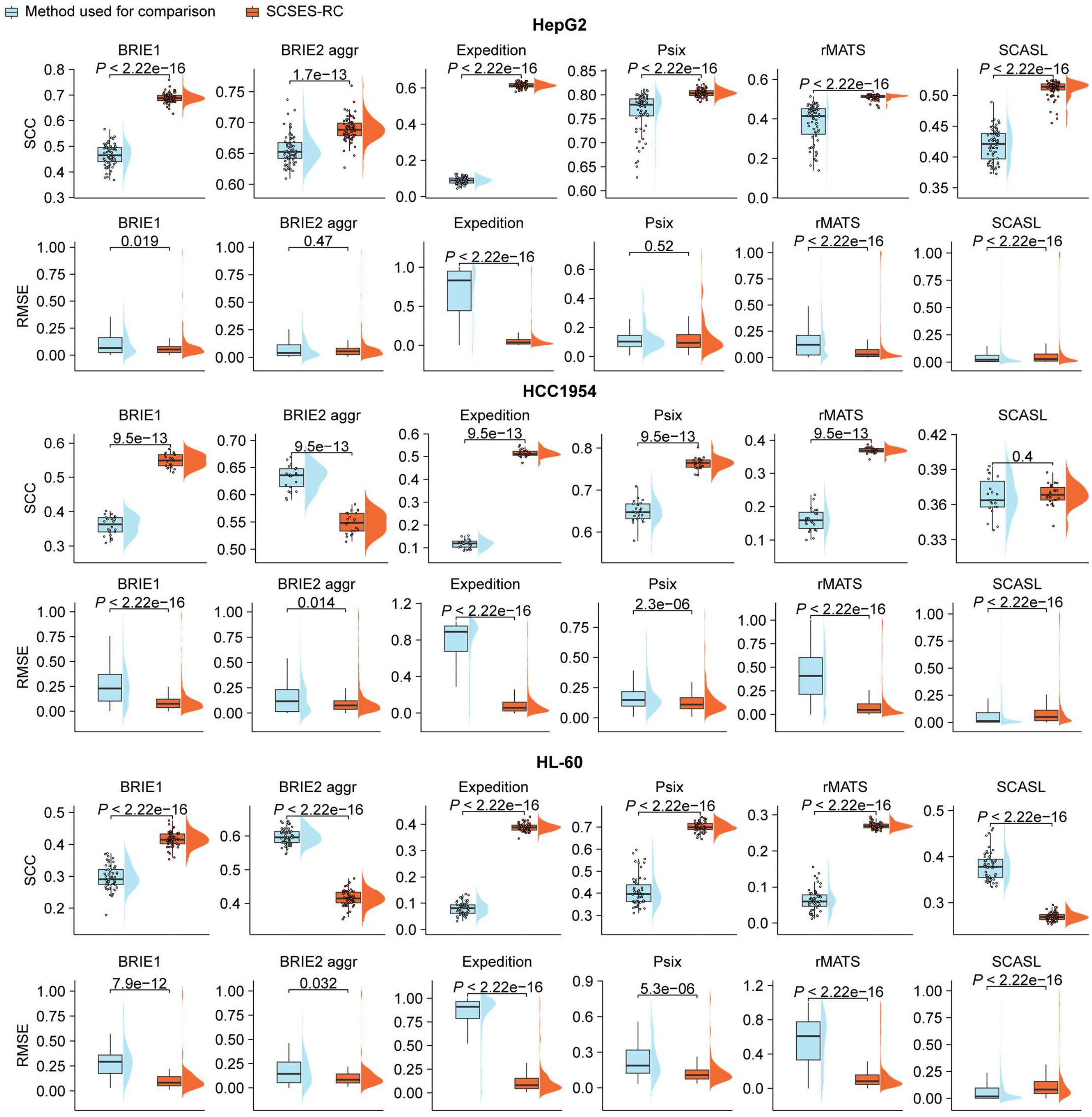

Supplementary Figure 13: Performance evaluation of SCSES-RC against existing algorithms on cell line scRNA-seq data. Raincloud plots showing the performance comparison between SCSES-RC and other algorithms in real scRNA-seq data of HepG2, HCC1954, and HL-60. The events detected by both SCSES and the compared algorithm are considered for each comparison. The SCC value refers to the correlation between estimated PSI values and benchmarks of all events in each cell. $N_{HCC1954}$ =22, N_{HCT116}=91, N_{HepG2} =68, $N_{HL\text{-}60}$=54. The RMSE is calculated between estimated PSI and benchmarks of an event among all cells. N_{BRIE1}=556, $N_{BRIE2\ aggr}$=556, $N_{Expedition}$=1,458, N_{Psix}=495, N_{rMATS}=10,313, N_{SCASL}=10,313. The boxes indicate median (center), Q25, and Q75 (bounds of box), the smallest value within 1.5 times interquartile range below Q25 and the largest value within 1.5 times interquartile range above Q75 (whiskers). The *P*-values are calculated by the Wilcoxon test (two-sided test). For pairwise comparisons, unadjusted *P*-values are reported.

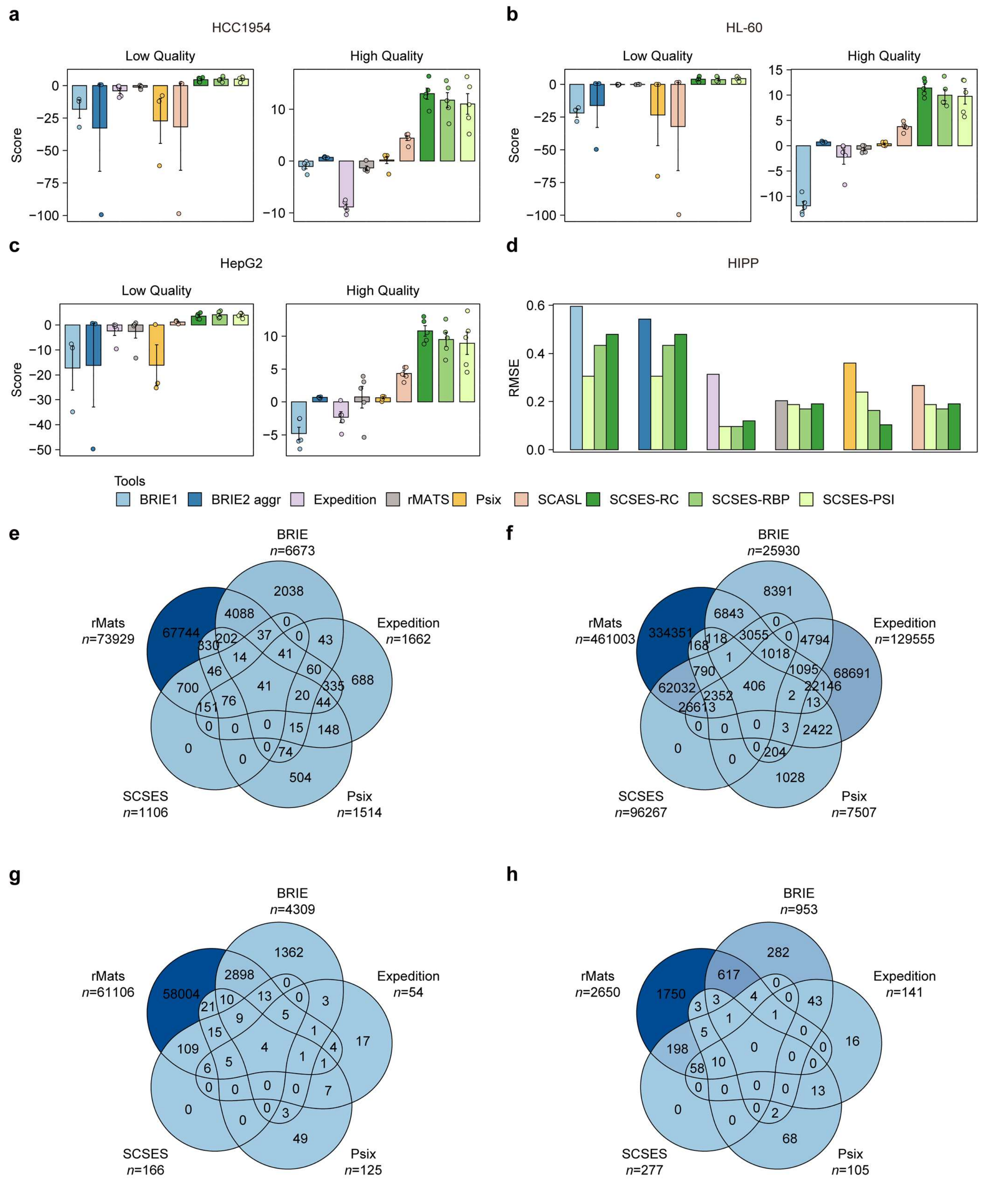

Supplementary Figure 14: Performance evaluation of SCSES against existing algorithms on synthetic datasets and Human Hippocampus dataset. (a,b,c) Bar plots showing the accuracy scores of different algorithms in HCC1954 (**a**), HL-60 (**b**), and HepG2 (**c**) synthetic datasets. The accuracy score is defined as the product of correlation between inference and benchmarks and AS events recall rate. Error bars represent the standard error of the mean from five independent replicates. (**d**). Bar plots comparing the performance of different methods on Human Hippocampus dataset. The RMSE is calculated between the estimated PSI values by different methods and benchmark values averaged on all cell-event pairs. (**e**) Venn diagram showing the event counts identified by different methods from the short-read data of the Ovarian Cancer dataset. (**f**) Venn diagram showing the high-confidence event–cell pair counts from the long-read data used as a benchmark in the Ovarian Cancer dataset. (**g**) Venn diagram showing the event counts identified by different methods from the short-read data of the Human Hippocampus dataset. (**h**) Venn

diagram showing the high-confidence event–cell pair counts from the long-read data used as a benchmark in the Human Hippocampus dataset.

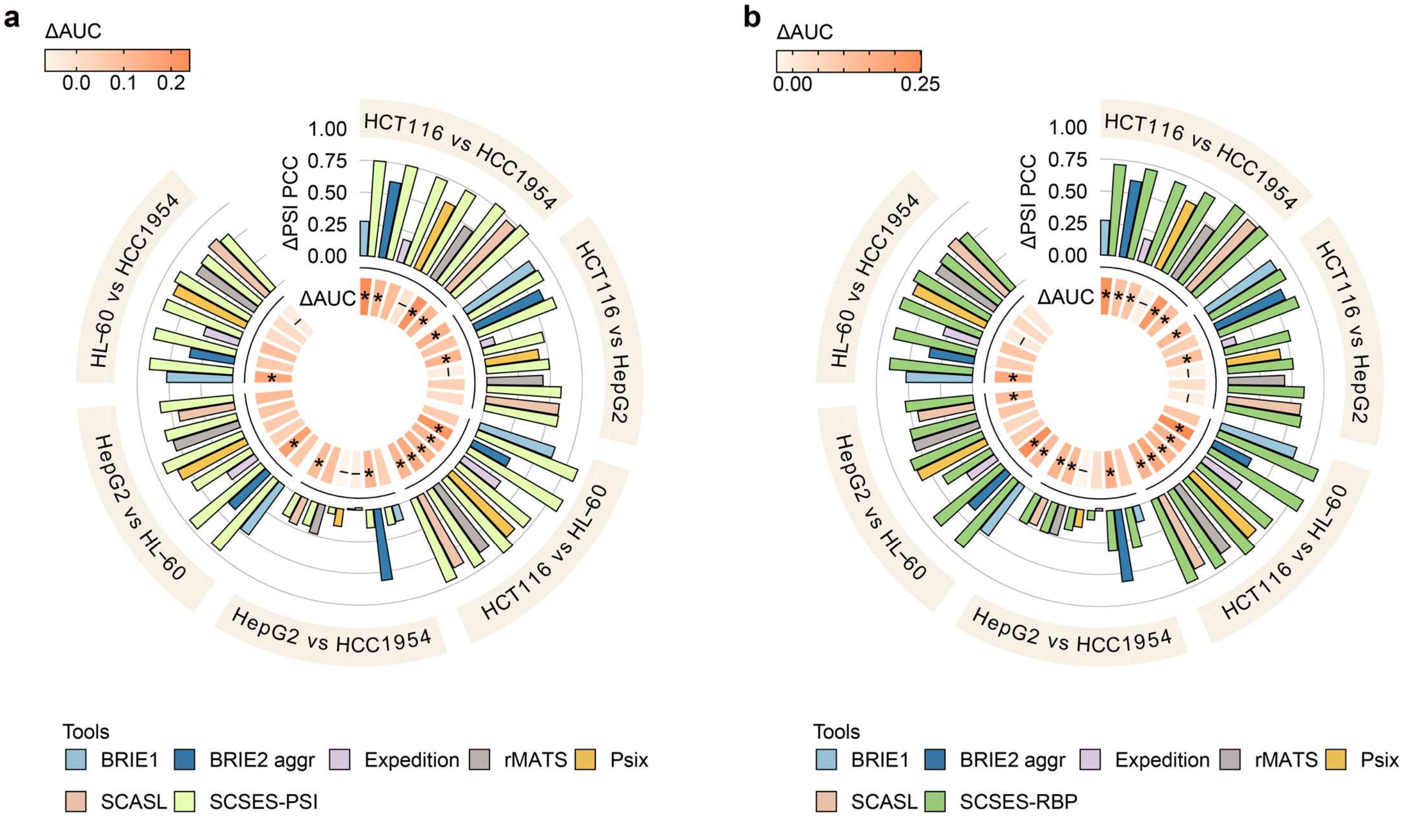

Supplementary Figure 15: Comparison of the detected DSEs between SCSES-PSI (a), SCSES-RBP (b) with other algorithms in the real datasets. Bar plot shows the SCC of ΔPSI in DSEs from the benchmark in each comparison group. The color of each bar represents different algorithms. The inner circle represents the difference of AUC for DSEs identification between SCSES and the compared algorithm. On the circle, *: $AUC_{SCSES} - AUC_{ref} > 0.1$, -: $AUC_{SCSES} - AUC_{ref} < 0$.

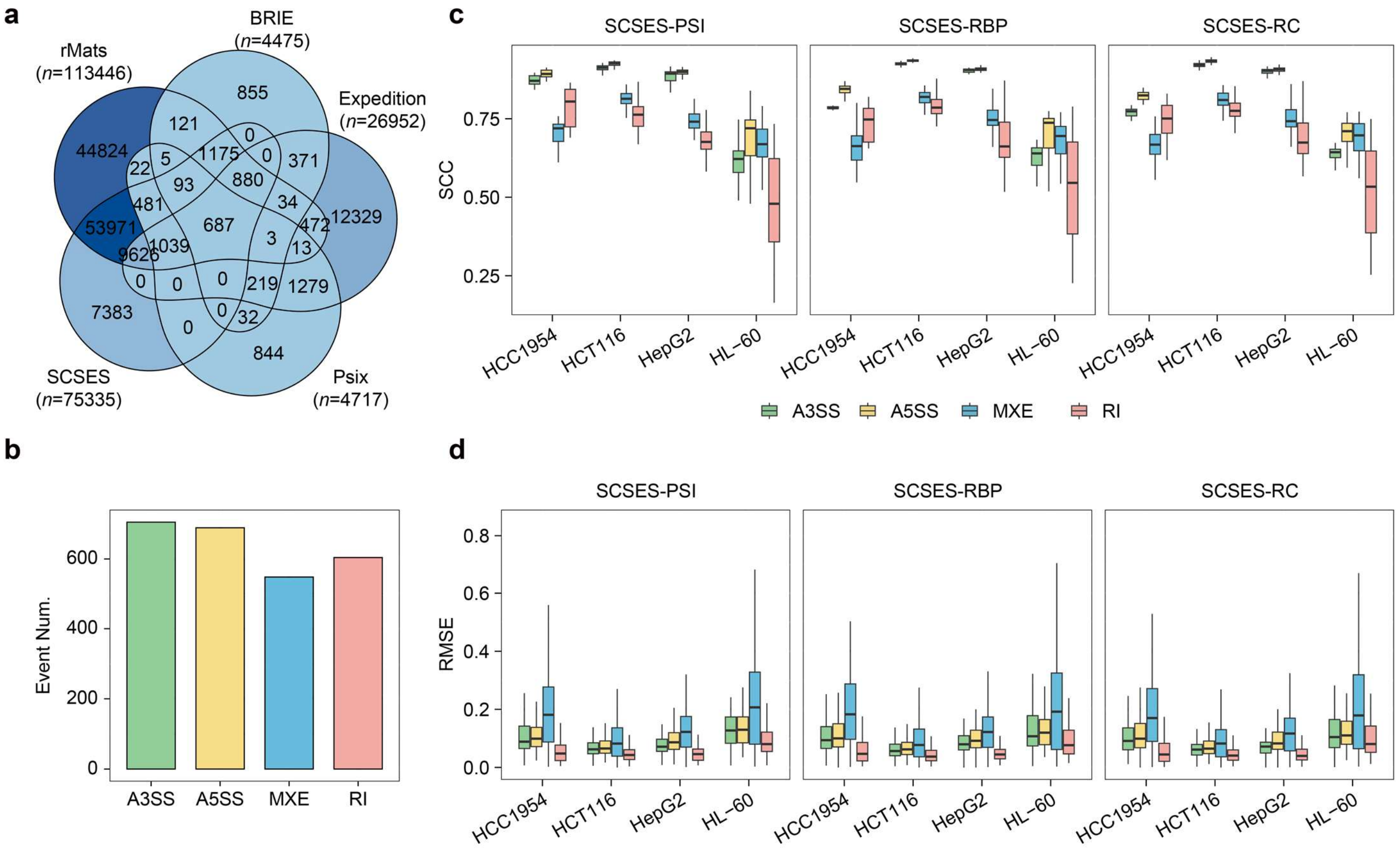

Supplementary Figure 16: SCSES can identify more events than other methods. (**a**). Venn diagram showing the event counts identified by different methods in real cell line datasets. (**b**). Bar plot showing the event counts of different event types except SE. (**c**). Box plots showing the performance of SCSES in real scRNA-seq data of HepG2, HCC1954, and HL-60. The SCC value refers to the correlation between estimated PSI values and benchmarks of different event types except SE in each cell. N_{HCC1954} =22, N_{HCT116}=91, N_{HepG2} =68, $N_{\text{HL-60}}$=54. The boxes indicate median (center), Q25, and Q75 (bounds of box), the smallest value within 1.5 times interquartile range below Q25 and the largest value within 1.5 times interquartile range above Q75 (whiskers). (**d**). The RMSE value was calculated between estimated PSI values and benchmarks of different event types except SE for each individual splicing event across all cells. The boxes indicate median (center), Q25, and Q75 (bounds of box), the smallest value within 1.5 times interquartile range below Q25 and the largest value within 1.5 times interquartile range above Q75 (whiskers).

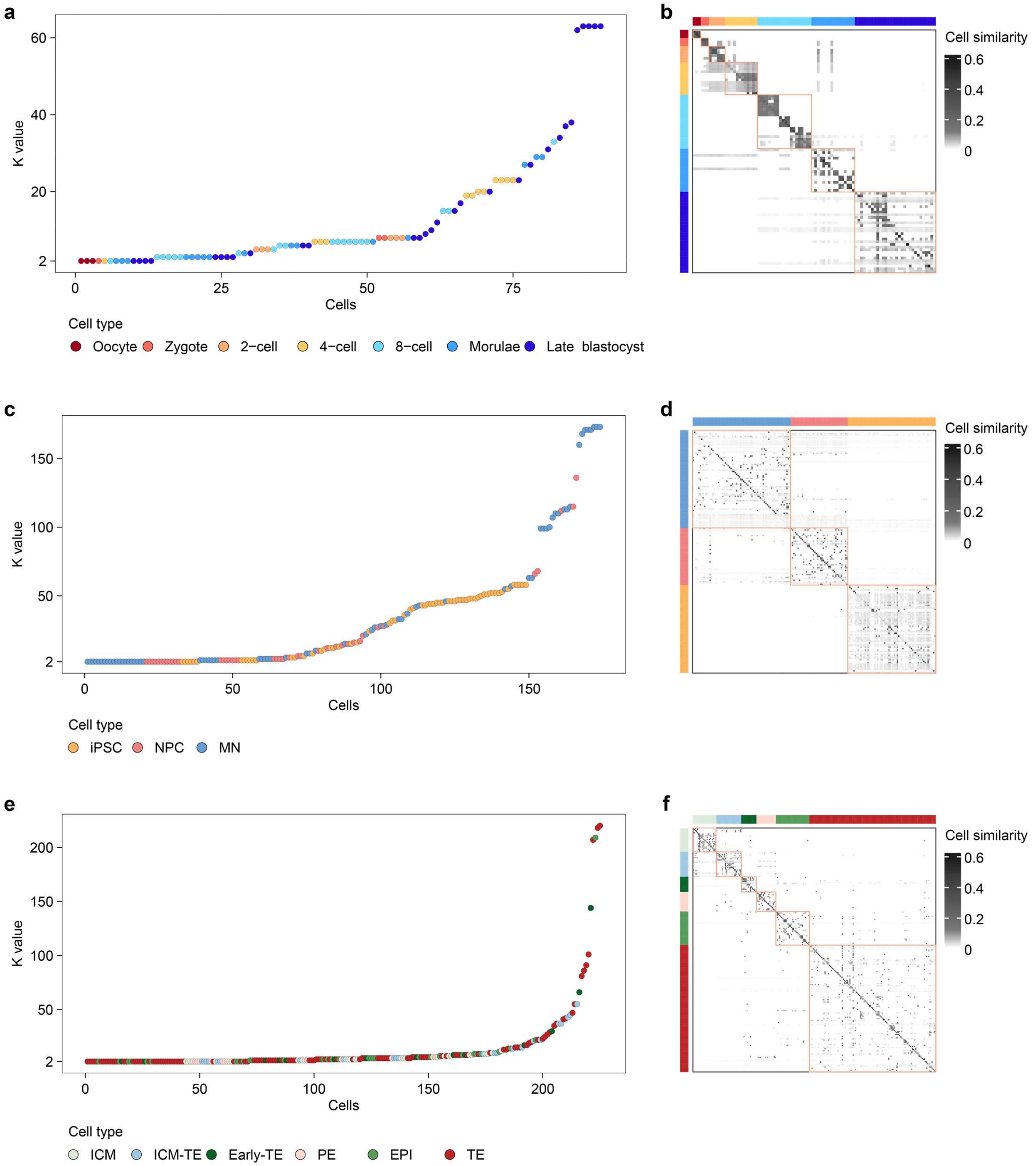

Supplementary Figure 17: Dynamic-K algorithm performance evaluation. (**a, c, e**) The final K value of each cell determined by dynamic-K algorithm in hEE (**a**), iPSC(**c**) and nPSC (**e**) down-sampling datasets. Colors of point represent different cell types. (**b, d, f**) Heatmap showing similarity scores between cells and their selected neighbors in hEE (**b**), iPSC (**d**) and nPSC (**f**) down-sampling datasets. Rows represent the target cells, and columns represent their selected neighbors. The similarities with non-selected cells are set to 0. N_{Oocyte}=3, N_{Zygote}=3, $N_{\mathrm{2\text{-}cell}}$=6, $N_{\mathrm{4\text{-}cell}}$=12, $N_{\mathrm{8\text{-}cell}}$=20, N_{Morulae}=16, $N_{\mathrm{Blastocyst}}$=30. N_{iPSC}=63, N_{NPC}=41, N_{MN}=70. N_{ICM}=22, $N_{ICM\text{-}TE}$=23, N_{PE}=14, $N_{Early\text{-}TE}$=18, N_{EPI}=31, N_{TE}=117.

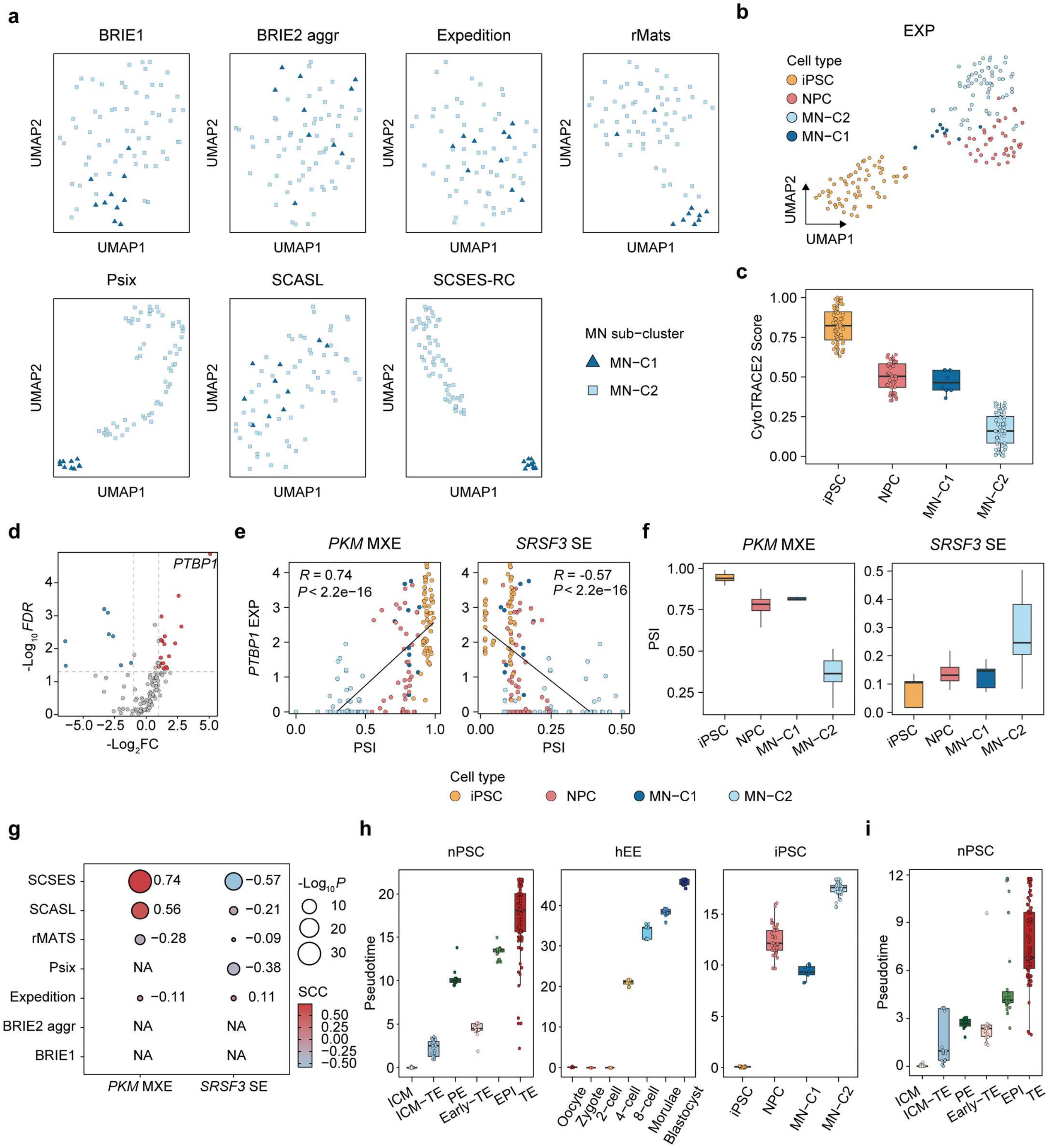

Supplementary Figure 18: **SCSES performance evaluation on biological implications.** (**a**) UMAP plots of 70 MN cells in iPSC test dataset by PSI values estimated by different methods. Colors and shapes of point represent different MN subgroups. $N_{MN\text{-}C1}$=10, $N_{MN\text{-}C2}$=60. (**b**) UMAP plots of 174 cells from the iPSC test dataset by gene expression. The colors indicate cell types. (**c**) The boxplot shows the distribution of stemness scores of in different cell groups. N_{iPSC}=63, N_{NPC}=41, $N_{MN\text{-}C1}$=10, $N_{MN\text{-}C2}$=60. (**d**). Volcano plot showing the differentially expressed RBPs between MN subclusters. The upregulated genes in the MN-C1 group are highlighted in red and the downregulated genes are highlighted in blue. (**e**). Scatter plots showing the expression of *PTBP1* (y-axis) versus the PSI of *PKM* (x-axis) (left panel), and *SRSF3* (x-axis) (right panel) in iPSC dataset. The correlation coefficient (R) and associated *P*-value are calculated by Spearman's correlation analysis. (**f**). Box plot showing the PSI of *PKM* and *SRSF3* among cell clusters in iPSC test dataset. (**g**). The correlation between *PTBP1* gene expression and PSI levels of two AS events (*PKM* MXE: left panel, *SRSF3* SE: right panel). The correlation coefficient (R) and associated *P*-value are calculated by Spearman's correlation analysis. (**h**). Box plot showing the consistence of pseudotime inferred by raw gene expression and real

differentiation stages in nPSC, iPSC and hEE datasets. (**i**). Box plot showing the consistence of pseudotime inferred by PSI values and real differentiation stages in nPSC dataset. All boxes indicate median (center), Q25, and Q75 (bounds of box), the smallest value within 1.5 times interquartile range below Q25 and the largest value within 1.5 times interquartile range above Q75 (whiskers). N_{ICM}=22, $N_{ICM\text{-}TE}$=23, N_{PE} =14, $N_{Early\text{-}TE}$=18, N_{EPI}=31, N_{TE}=117.

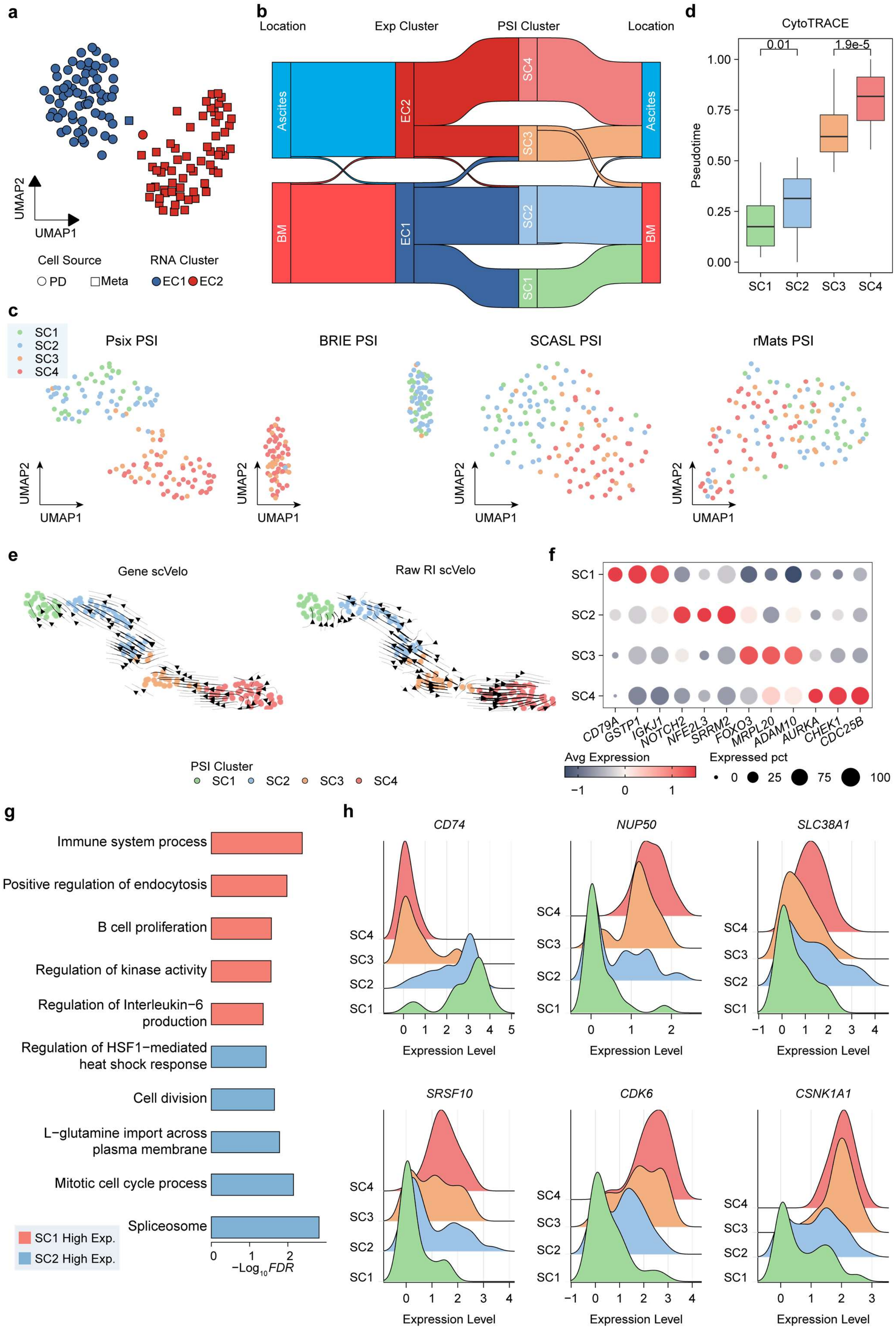
a
UMAP2
UMAP1
Cell Source
PD
Meta
RNA Cluster
EC1
EC2
b
Location
Exp Cluster
PSI Cluster
Location
Ascites
BM
EC2
EC1
SC4
SC3
SC2
SC1
d
CytoTRACE
Pseudotime
0.01
1.9e-5
1.00
0.75
0.50
0.25
0.00
SC1
SC2
SC3
SC4
c
SC1
SC2
SC3
SC4
Psix PSI
BRIE PSI
SCASL PSI
rMats PSI
e
Gene scVelo
Raw RI scVelo
PSI Cluster
SC1 SC2 SC3 SC4
f
CD79A GSTP1 IGKJ1 NOTCH2 NFE2L3 SRRM2 FOXO3 MRPL20 ADAM10 AURKA CHEK1 CDC25B
Avg Expression
−1 0 1
Expressed pct
0 25 75 100
g
Immune system process
Positive regulation of endocytosis
B cell proliferation
Regulation of kinase activity
Regulation of Interleukin−6 production
Regulation of HSF1−mediated heat shock response
Cell division
L−glutamine import across plasma membrane
Mitotic cell cycle process
Spliceosome
SC1 High Exp.
SC2 High Exp.
−Log10FDR
h
CD74
NUP50
SLC38A1
SRSF10
CDK6
CSNK1A1
Expression Level

Supplementary Figure 19: Analysis of the multiple myeloma dataset. (**a**). UMAP plots showing the cell clusters by gene expression (*N*=127). Colors represent the gene expression clusters, and shapes represent the source of samplings. PD: primary diagnosis, Meta: metastasis. (**b**). Sankey diagram showing the relationships among cell sampling locations, gene expression clusters and PSI clusters. (**c**). UMAP plots showing the cell projections of 127 MM cells using different AS inferring algorithms. Colors represent the cell clusters defined by SCSES. (**d**). box plot showing the relationship between PSI clusters and pseudotime estimated by CytoTRACE. N_{SC1}=25, N_{SC2}=38, N_{SC3}=23, N_{SC4}=41. The *P*-values are calculated by the Wilcoxon test (two-sided test) without any adjustments. The boxes indicate median (center), Q25, and Q75 (bounds of box), the smallest value within 1.5 times interquartile range below Q25 and the largest value within 1.5 times interquartile range above Q75 (whiskers). (**e**). RNA velocity estimated by original gene read counts (left panel) and raw junction counts of RI events (right panel) with scVelo. (**f**). Marker genes of SC1-SC4 clusters, omitting the DEGs between cell sampling locations. (**g**). The function enrichment analysis of DEGs between SC1 and SC2. (**h**). Ridge plots showing the expression of representative DEGs between SC1 and SC2.

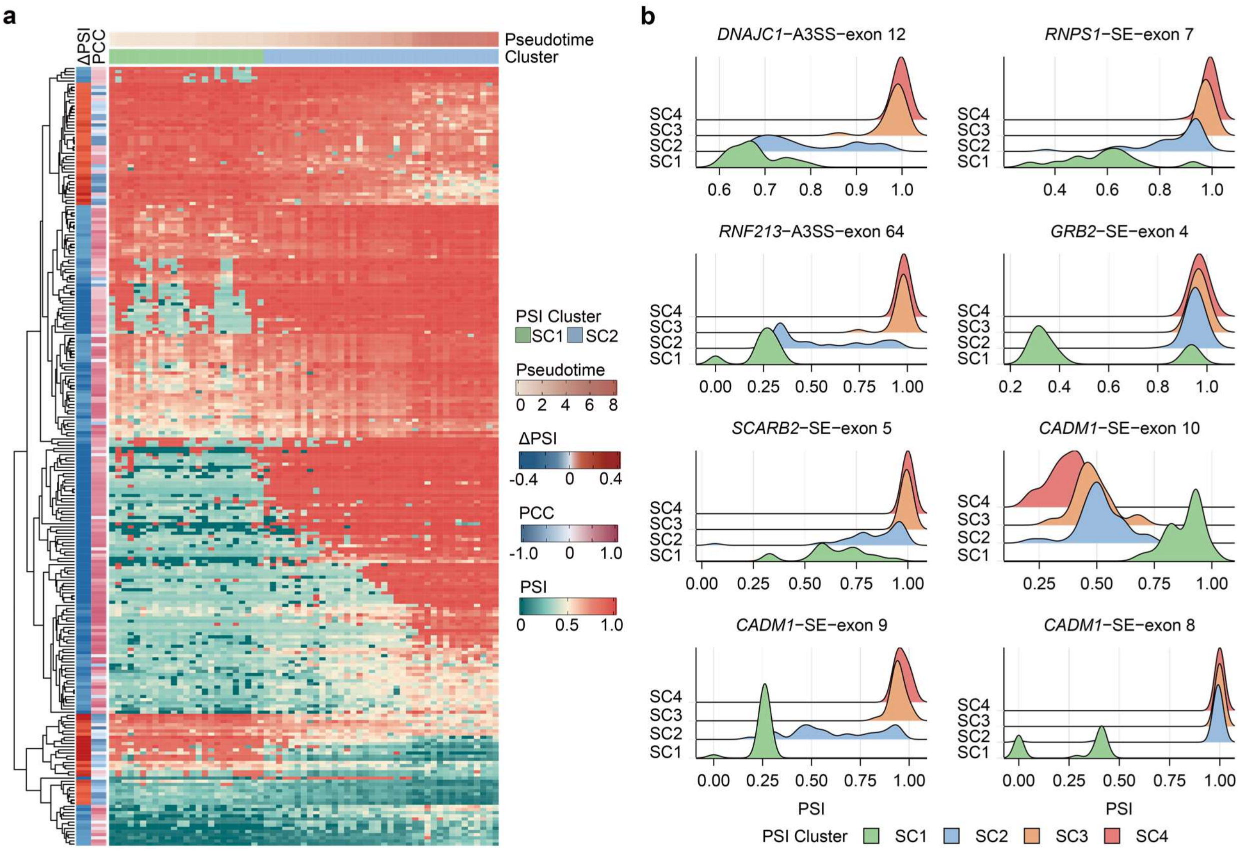

Supplementary Figure 20: Analysis of DSEs between SC1 and SC2. (**a**) Heatmap showing the splicing changes of DSEs between SC1 and SC2, while the overall expression of the genes harboring these DSEs are not differentially changes. ΔPSI represents the difference of averaged splicing levels in SC1 and SC2. PCC represents the correlation between PSI and pseudotime in all cells. (**b**) Ridge plots showing the PSI levels of representative DSEs between SC1 and SC2, whose target gene are not differentially expressed.

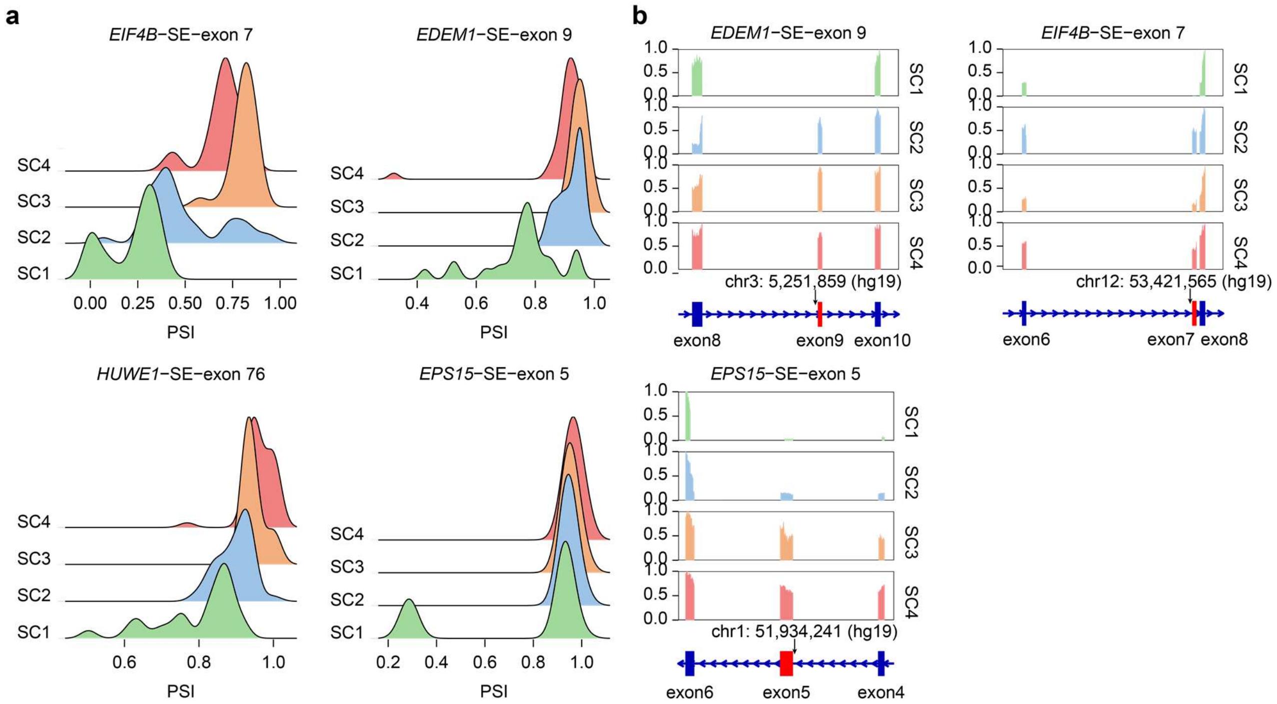

Supplementary Figure 21: Examples of DSEs associated with BTZ resistance. (**a**). Ridge plots showing the PSI levels of representative DSEs between SC1 and SC2, whose gene are reported to be associated with BTZ resistance. (**b**) Exon read coverage of three representative events associated with BTZ-resistance genes by merging the reads from the same cell group. Alternative exons are highlighted in red.

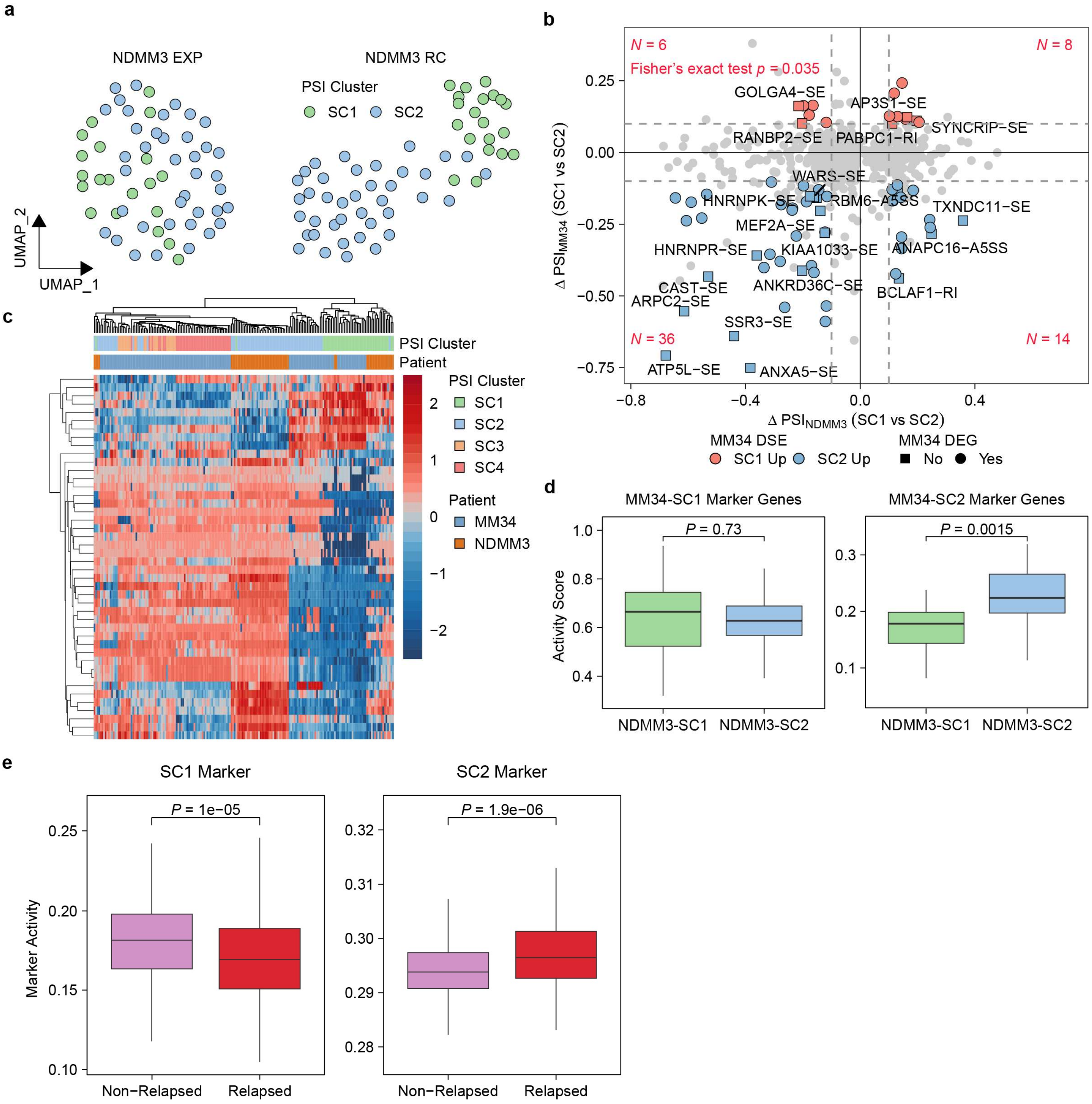

Supplementary Figure 22: Orthogonal validation of splicing-based subclusters in multiple myeloma patients. (**a**) UMAP plots showing cell projections for 59 NDMM3 cells based on gene expression profile (left) and splicing profile (right). Colors indicate the two sub-clusters by splicing profile. (**b**) Scatter plot showing the direction of commonly differentially spliced events (DSEs) in NDMM3 and MM34. Commonly differentially spliced events are highlighted by colors, with shapes indicating whether the corresponding genes were differentially expressed between SC1 and SC2 of MM34. (**c**) Unsupervised hierarchical clustering and heatmap of all cells from NDMM3 and MM4 by the 44 DSEs with the same splicing direction between SC1 to SC2 in (b). (**d**) Box plots comparing marker gene activities of MM34 SC1 and SC2 subclusters in subclusters identified in NDMM3. N_{SC1}=20, N_{SC2}=39. The *P*-values are calculated by the Wilcoxon test (two-sided test) without any adjustments. The boxes indicate median (center), Q25, and Q75 (bounds of box), the smallest value within 1.5 times interquartile range below Q25 and the largest value within 1.5 times interquartile range above Q75 (whiskers). (**e**) Box plots comparing MM34-SC1 and MM34-SC2 marker gene activities in primary diagnosis samples, stratified by patient relapse status. The *P*-values are calculated by the Wilcoxon test (two-sided test) without any adjustments. The boxes indicate median (center), Q25, and Q75 (bounds of box), the

smallest value within 1.5 times interquartile range below Q25 and the largest value within 1.5 times interquartile range above Q75 (whiskers).

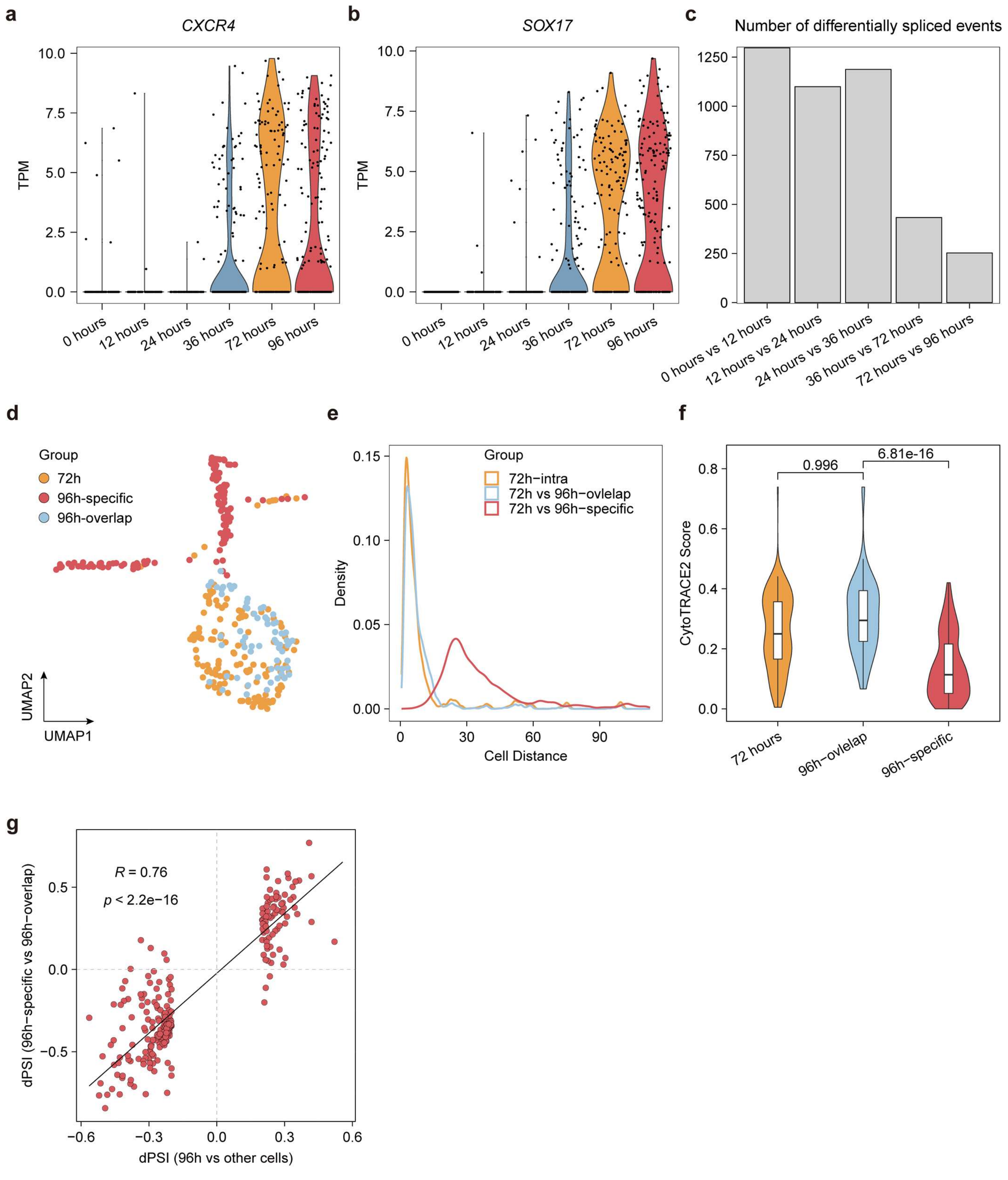

Supplementary Figure 23: Correlation analysis between 72h- and 96h-cells in embryo stem cell data. (**a**). Violin plot showing the expression of CXCR4 among time points. $N_{0\ hours}$=92, $N_{12\ hours}$=102, $N_{24\ hours}$=66, $N_{36\ hours}$=172, $N_{72\ hours}$=138, $N_{96\ hours}$=188. (**b**). Violin plot showing the expression of SOX17 among time points. (**c**). Bar plot showing number of differentially spliced events between each pair of neighboring time points. (**d**). UMAP plots of 326 cells from 72 and 96 hours, colored by cell subpopulations. $N_{96h\text{-}specific}$ =123, $N_{96h\text{-}overlap}$=65. (**e**). Distribution plots showing pairwise cell distances for three comparisons: intrapopulation distances within 72h cells, interpopulation distances between 72h and 96h-specific cells, and between 72h and 96h-overlapped cells. (**f**). CytoTrace score for cells in the three subpopulations. The boxes indicate median (center), Q25, and Q75 (bounds of box), the smallest value within 1.5 times interquartile range below Q25 and the largest value within 1.5 times interquartile range above Q75 (whiskers). Wilcoxon rank-sum test were used to evaluate the statistical significance between different groups (one-sided test, using FDR

correction to adjust for multiple comparisons). (**g**). Scatter plot comparing splicing changes of differentially spliced events identified between 96h and other time points in two contexts: 96h cells versus other cells (x-axis) and 96h-specific cells versus 96h-overlapped cells (y-axis). The correlation coefficient (R) and associated *P*-value are calculated by Spearman correlation analysis.

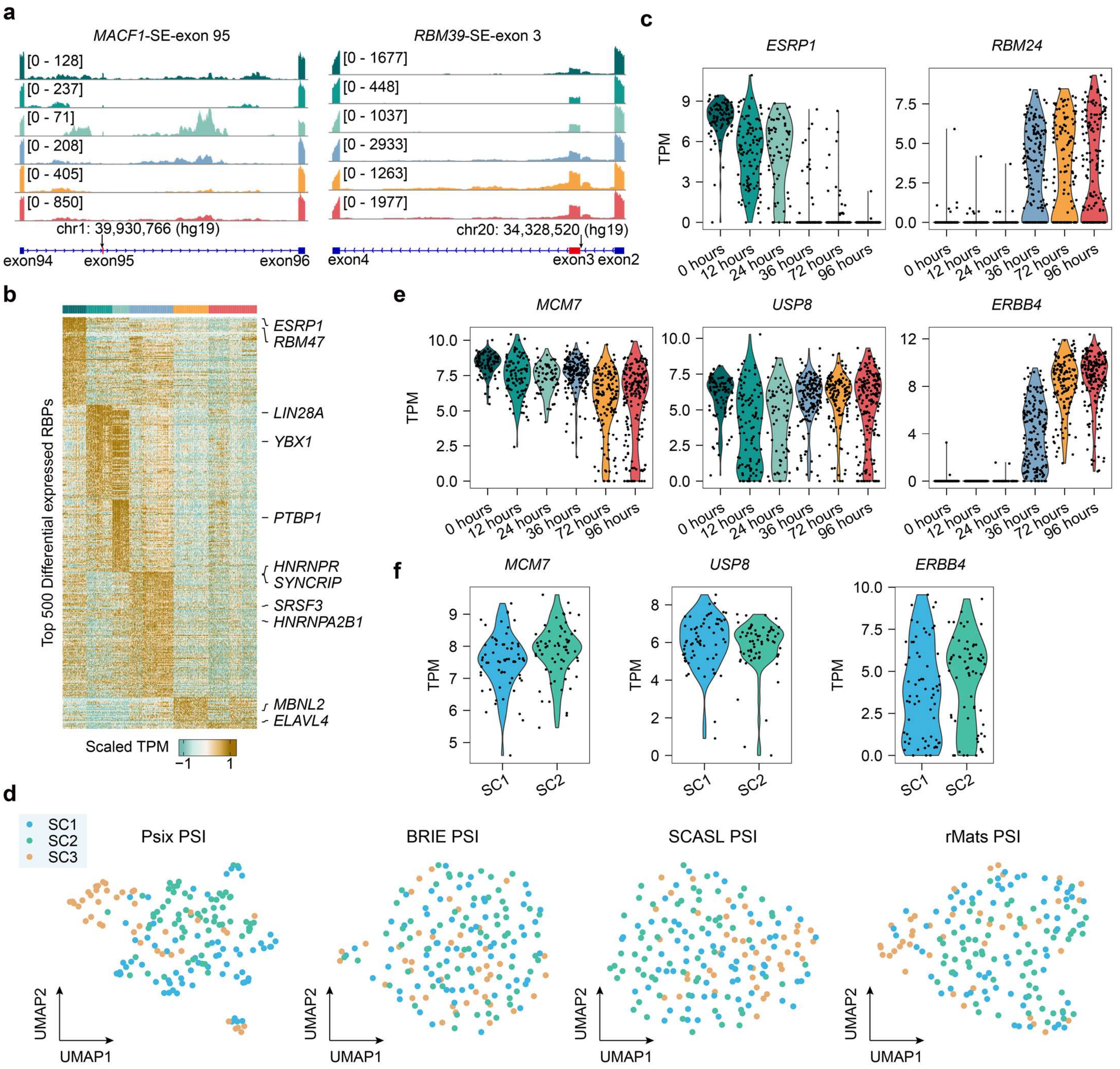

Supplementary Figure 24: Analysis of the hES dataset. (**a**). Read coverage showing the inclusion of *MACF1* exon 95 and *RBM39* exon 3 on the pseudobulk of each time points. Alternative exons are highlighted in red. (**b**). Heatmap showing the top 500 uniquely expressed RPBs at specific time points. (**c**). The violin plots showing the dynamic expression of *ESRP1*, *RBM47*, and *RMB24* at different time points. (**d**) UMAP plots showing the cell projections of developing 36h cells using different AS inferring algorithms (N=172). Colors represent the cell clusters defined by SCSES. (**e**). The violin plots showing the dynamic expression of *MCM7*, *USP8*, and *ERBB4* at different time points. (**f**). The violin plots showing the differential expression of *MCM7*, *USP8*, and *ERBB4* in SC1 and SC2 at 36h. N_{SC1}=66, N_{SC2}=65.

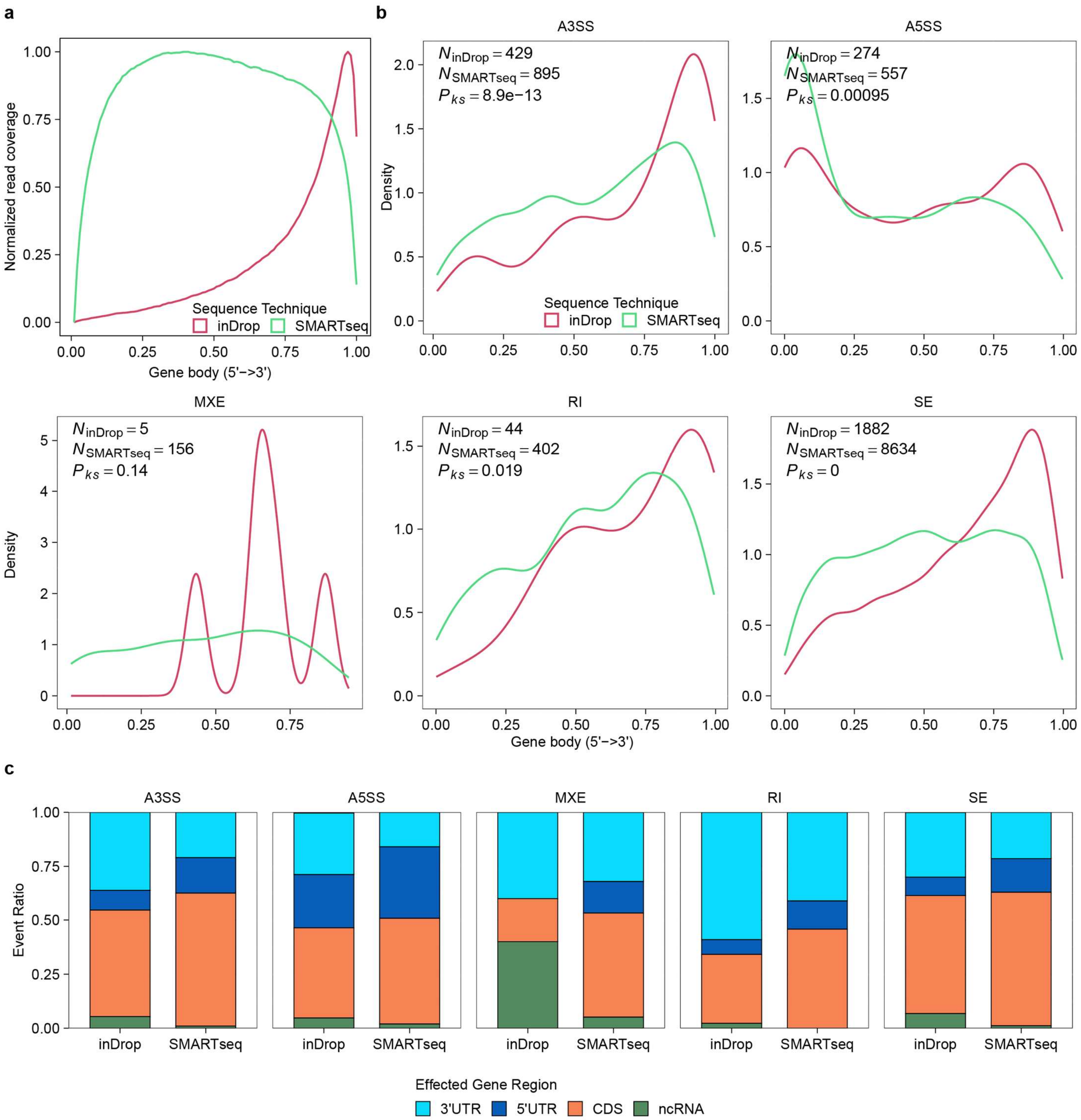

Supplementary Figure 25: Comparison of sequencing read distributions between inDrop and SMART-seq datasets. (**a**) Density plot showing the distribution of sequencing reads along gene bodies, from transcript start sites to transcript termination sites. (**b**) Density plot showing the relative distance of AS regions to the transcript start site, stratified by AS event types. ks: Kolmogorov-Smirnov Test. (**c**) Bar plots showing the percentage of AS events in each gene region (ncRNA, 5'UTR, CDS, 3'UTR), stratified by AS types. ncRNA: the genes harboring the events is non-coding genes. 3'UTR: either the upstream or downstream exon of the alternative region contains 3'UTR. 5'UTR: either the upstream or downstream exon of the alternative region contains 5'UTR. CDS: the upstream and downstream exon of the alternative region contain only coding sequences.

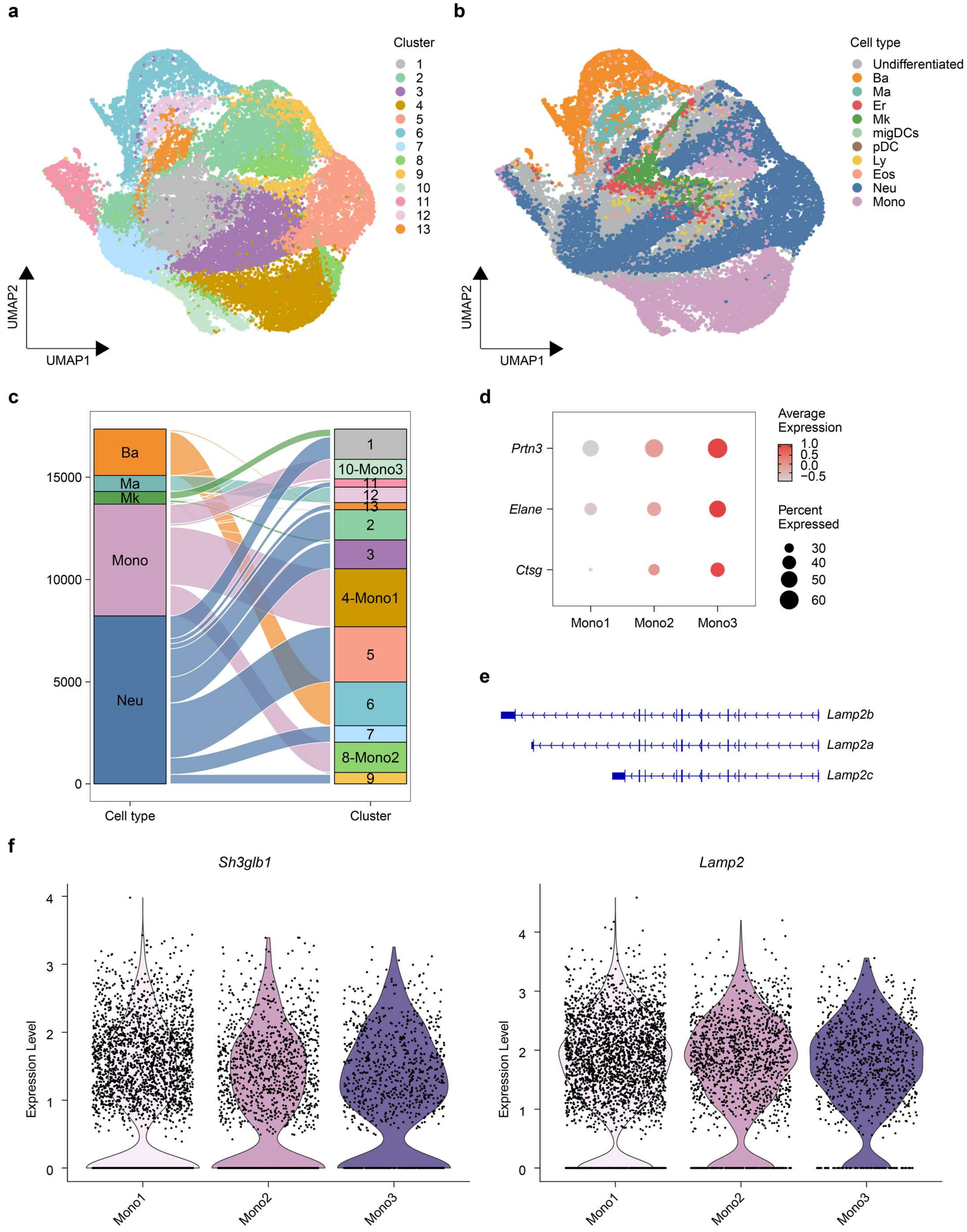

Supplementary Figure 26: Analysis of the induced HSC differentiation dataset. (**a**,**b**). UMAP plot showing 30,686 mouse HSCs, colored by Leiden clusters (**a**) and cell annotation (**b**). Eos: eosinophils. Ly: lymphoid precursors. Er: erythrocytes. Ma: mast cells. Ba: basophils. pDC: plasmocytoid dendritic cells. migDCs: Ccr7+ migratory DCs. Mk:

megakaryocytes. Neu: neutrophils. Mono: monocytes. (**c**) Sankey diagram showing the relationship between cell types and Leiden clusters. (**d**). Bubble heatmap showing the expression of *Prtn3*, *Elane*, and *Ctsg* in the monocyte sub-clusters. (**e**). The gene structures of *Lamp2* isoforms with alternative last exons. (**f**). Violin plots showing the expression of *Sh3glb1* (left panel) and *Lamp2* (right panel) among 3 subclusters of monocytes. N_{Mono1}=2828, N_{Mono2}=1477, N_{Mono3}=951.

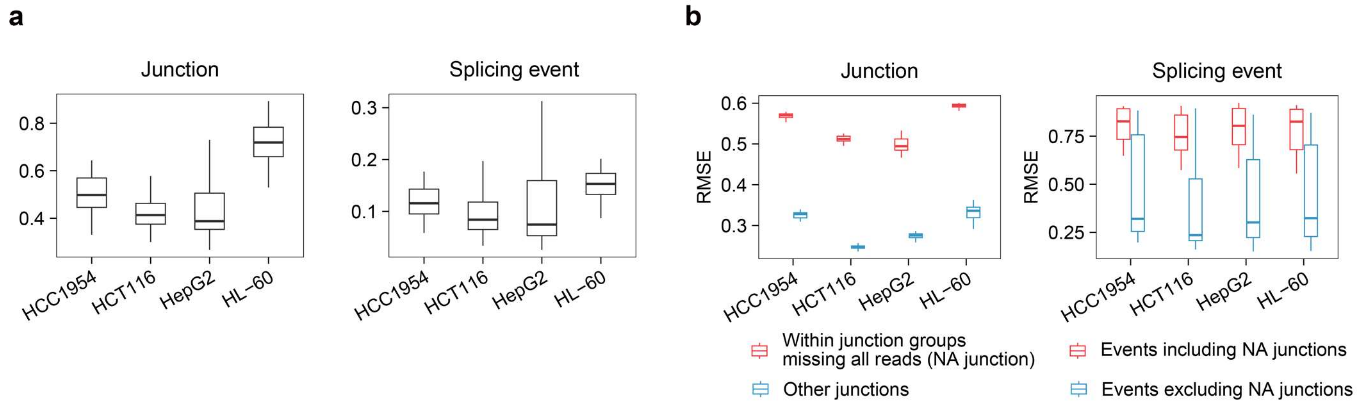

Supplementary Figure 27: Analysis of the missing values in junction groups and splicing events. (**a**). **left panel**: box plot showing the percentages of junction groups missing all junction read within a cell based on the AS probability matrix estimated by SCASL from cell line datasets. right panel: box plot showing the percentage of splicing events containing junctions from junction group missing all junction read in a cell from the cell line datasets. The total set of events are detected by SCSES. $N_{HCC1954}$ =22, N_{HCT116}=91, N_{HepG2} =68, $N_{\text{HL-60}}$=54. (b). left panel: box plot showing the RMSE of junction probabilities between benchmarks and SCSAL results grouped by if junction groups miss all junction reads from real cell line datasets. right panel: box plot showing the RMSE of event PSI values between benchmarks and SCSAL results grouped by if events contain junctions from the junction group missing all junction reads in real cell line datasets. The total set of events are detected by SCSES, and the PSI values are transformed from SCSAL results. All boxes indicate median (center), Q25, and Q75 (bounds of box), the smallest value within 1.5 times interquartile range below Q25 and the largest value within 1.5 times interquartile range above Q75 (whiskers). $N_{HCC1954}$ =22, N_{HCT116}=91, N_{HepG2} =68, $N_{\text{HL-60}}$=54.

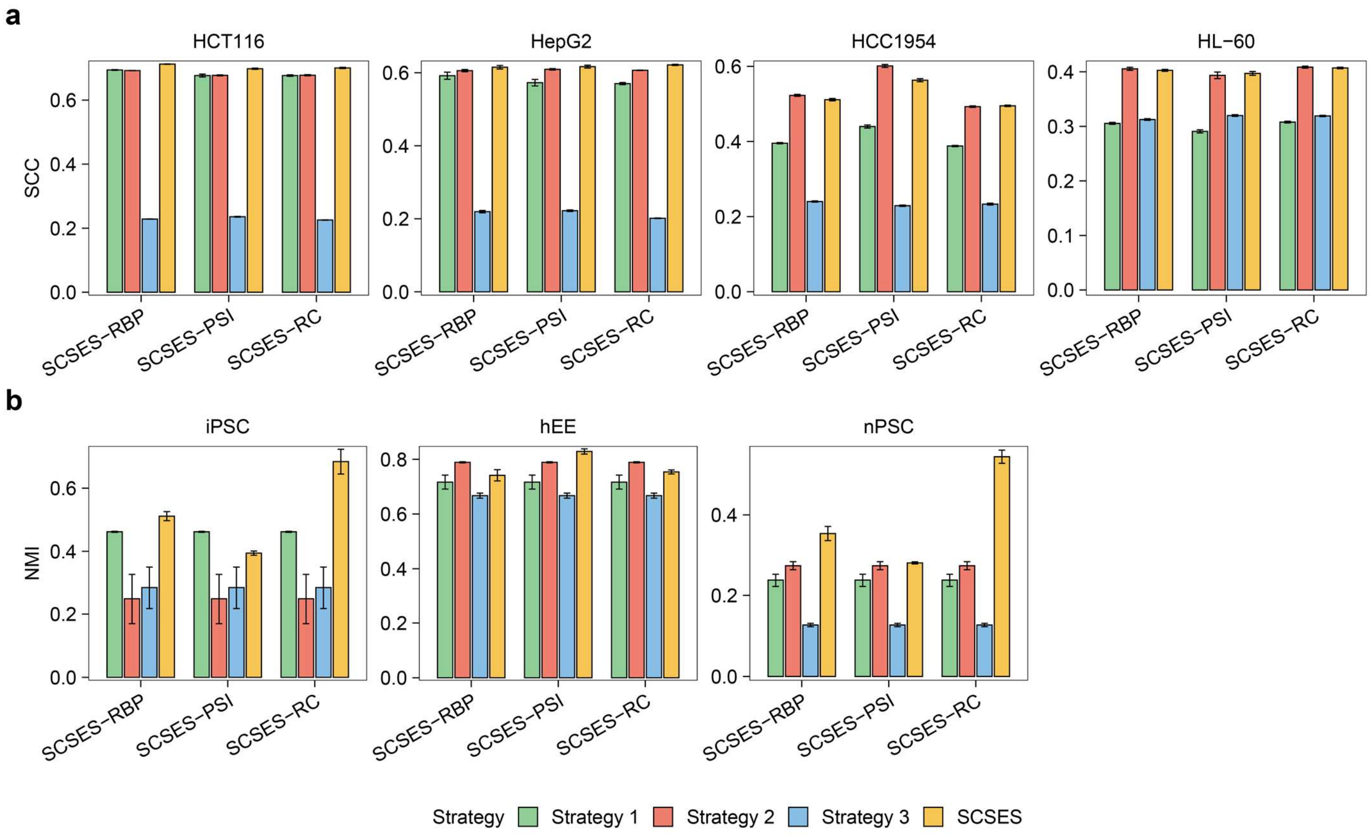

Supplementary Figure 28: Ablation study evaluating the contribution of different imputation strategy integrations. (**a**) Spearman correlation coefficients (SCC) between estimated and benchmark PSI values for all events across cells, comparing different imputation strategies. Error bars represent the standard error of the mean in all cells across cell lines. $N_{HCC1954}$ =22, N_{HCT116}=91, N_{HepG2} =68, $N_{\text{HL-60}}$=54. (**b**) Normalized Mutual Information (NMI) scores assessing clustering performance in down-sampling datasets of iPSC, hEE, and nPSC for each strategy. Error bars represent the standard error of the mean from three independent replicates.

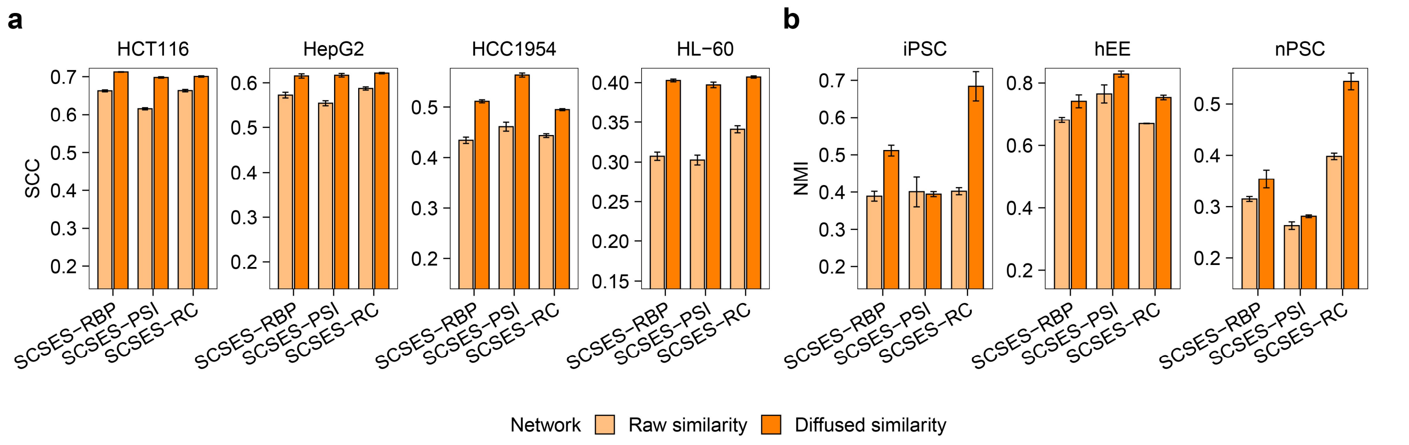

Supplementary Figure 29: Ablation study evaluating the impact of similarity diffusion on imputation performance. (**a**) Comparison of PSI estimation accuracy (Spearman correlation coefficients, SCC) between estimated and benchmark values for raw versus diffused similarity networks. Error bars represent the standard error of the mean in all cells across cell lines. $N_{HCC1954}$ =22, N_{HCT116}=91, N_{HepG2} =68, $N_{\text{HL-60}}$=54. (**b**) Clustering performance (Normalized Mutual Information, NMI) in down-sampling datasets (iPSC, hEE, nPSC) using raw versus diffused similarity approaches. Error bars represent the standard error of the mean from three independent replicates.

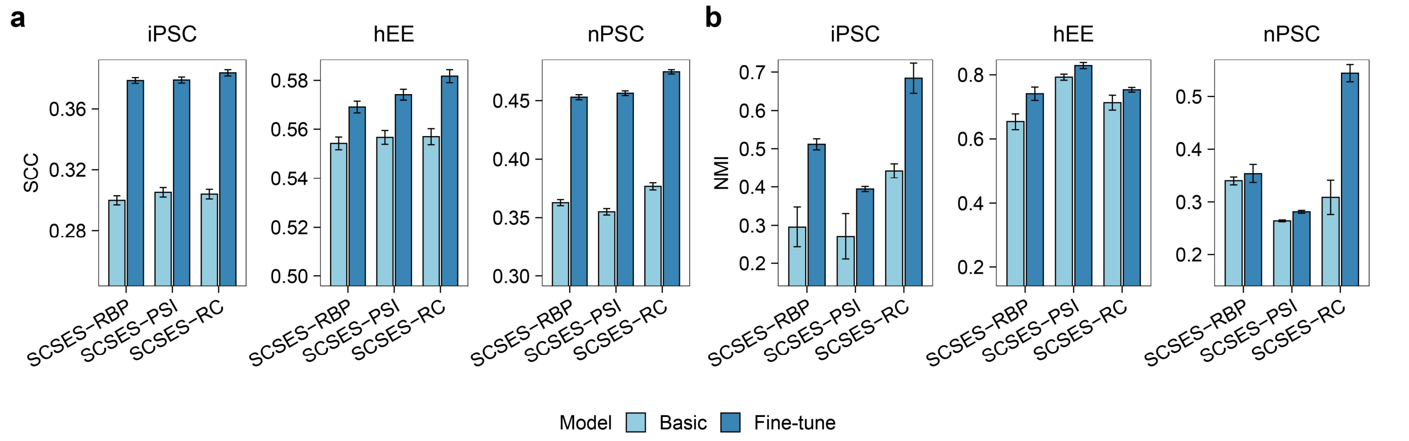

Supplementary Figure 30: Ablation study evaluating the impact of model fine-tuning on splicing imputation performance. (**a**) Comparison of PSI estimation accuracy (Spearman correlation coefficients, SCC) between gene-expression-derived pseudotime and PSI-derived pseudotime for pre-trained versus fine-tuned models. Error bars represent the standard error of the mean in all cells of different datasets. (**b**) Clustering performance (Normalized Mutual Information, NMI) in down-sampling stem cell datasets (iPSC, hEE, nPSC) comparing pre-trained and fine-tuned models. Error bars represent the standard error of the mean from three independent replicates.

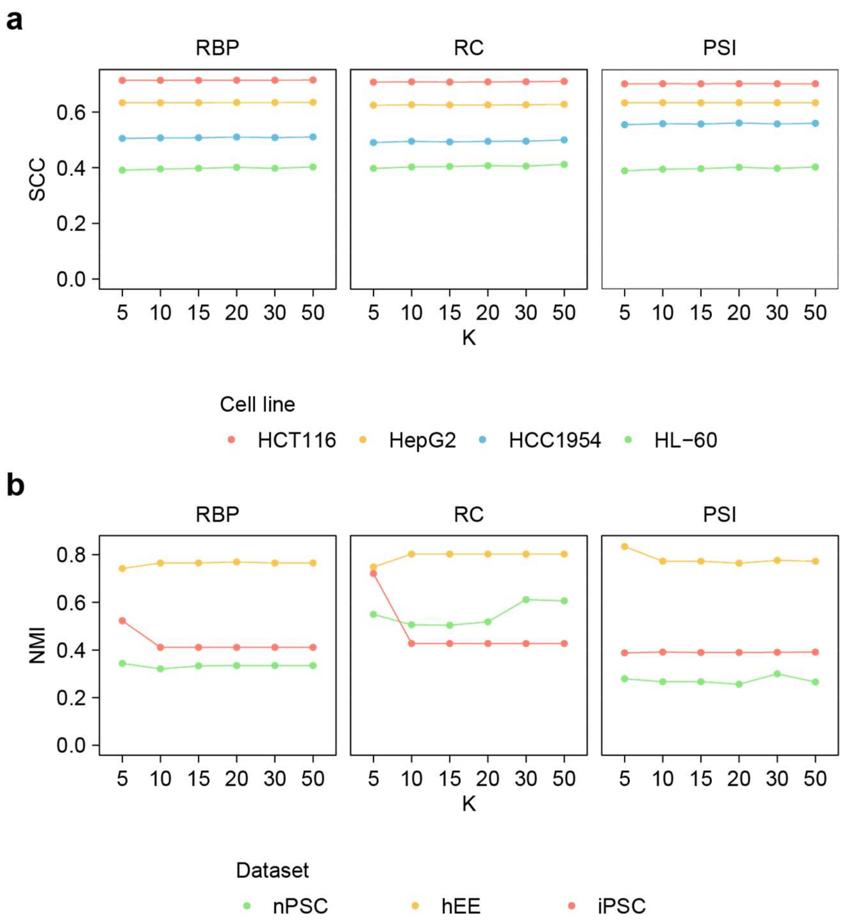

Supplementary Figure 31: Performance evaluation of event similarity computation across different K values. (**a**) SCC among cells in four cell line datasets. The points indicate median SCC in all cells across cell lines. (**b**) NMI in down-sampling datasets. The points indicate median NMI from three independent replicates.

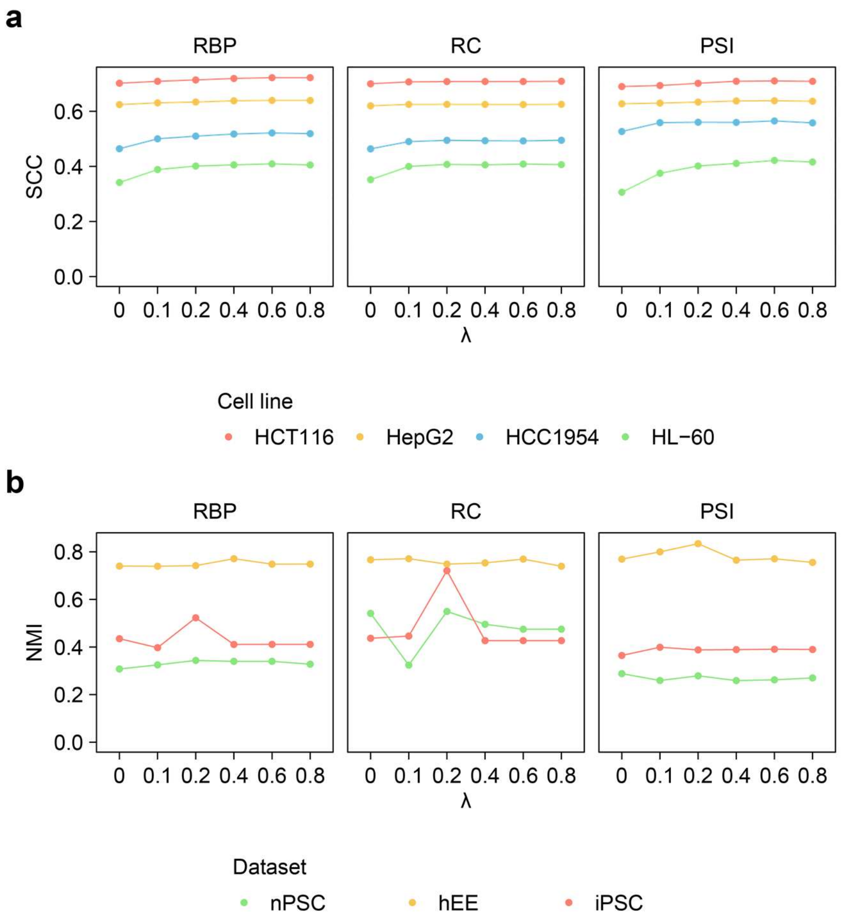

Supplementary Figure 32: Performance evaluation of Random Walk with Restart algorithm under varying restart probabilities (λ). (**a**) SCC among cells in four cell line datasets. The points indicate median SCC in all cells across cell lines. (**b**) NMI in down-sampling datasets. The points indicate median NMI from three independent replicates.

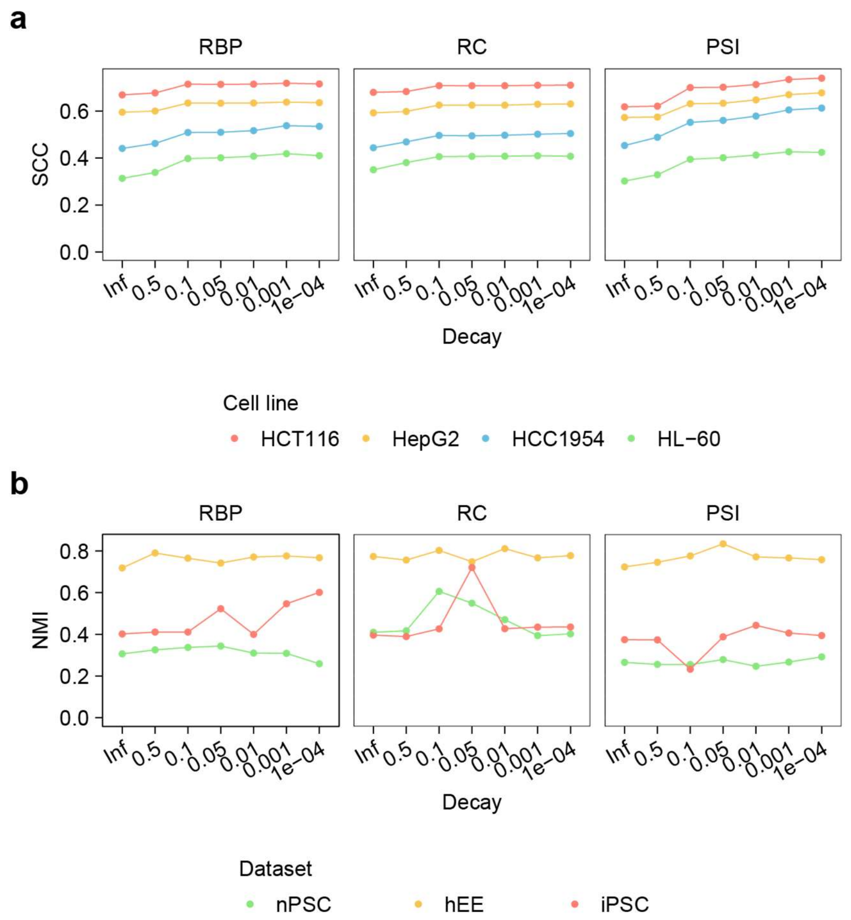

Supplementary Figure 33: Performance evaluation of random walk algorithm under different convergence thresholds (Decay). (**a**) SCC among cells in four cell line datasets. The points indicate median SCC in all cells across cell lines. (**b**) NMI in down-sampling datasets. Note: "Inf" indicates no diffusion procedure was applied. The points indicate median NMI from three independent replicates.

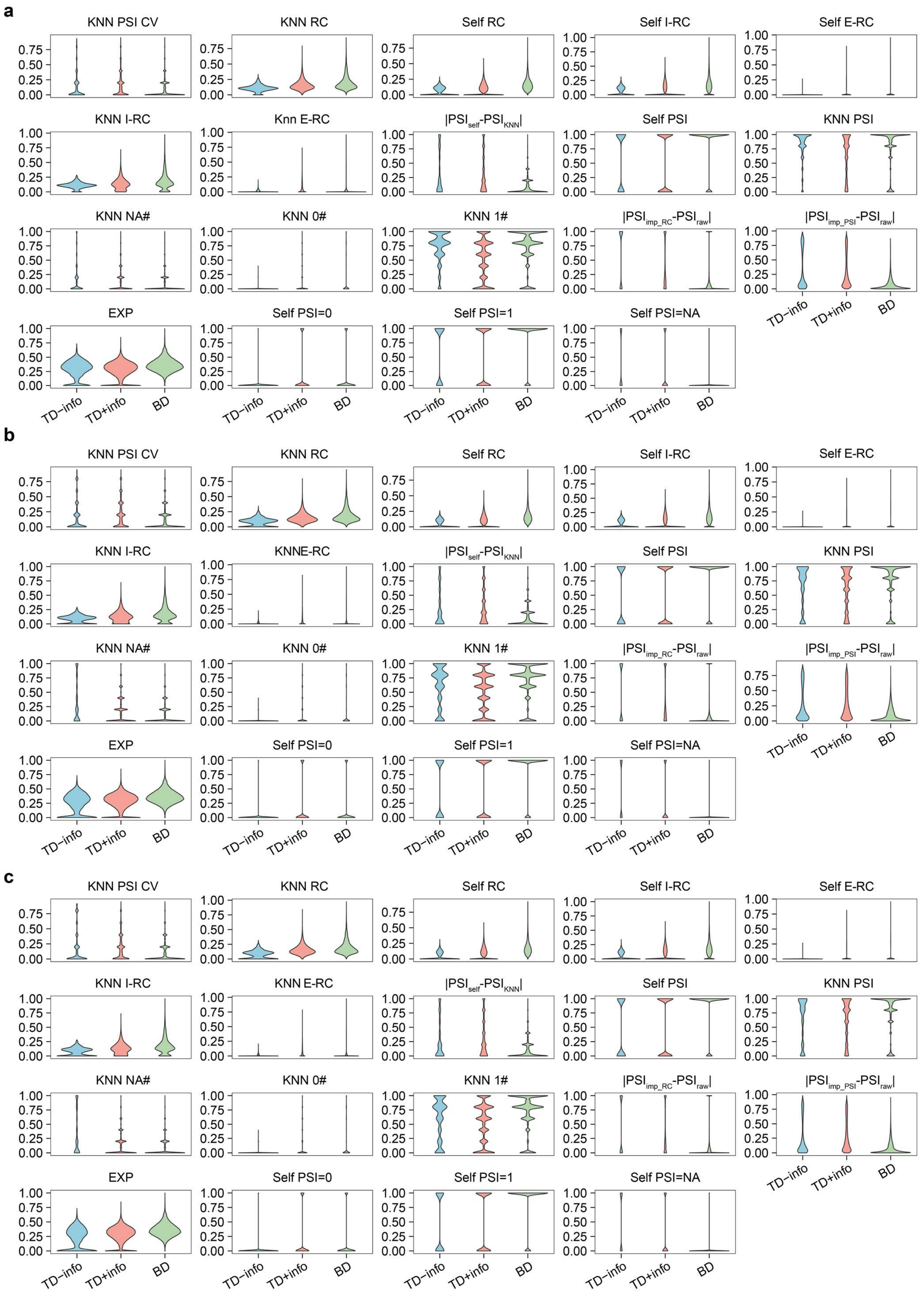

Supplementary Figure 34: Differential feature distributions across scenarios. Violin plots showing the 19 features used in Model1 and Model2 exhibit different levels in different scenarios, no matter cell similarities are estimated by

RBP. $N_{TD\text{-}info}$ =13,056, $N_{TD+info}$=378,633, N_{BD} =423,441 (**a**), PSI $N_{TD\text{-}info}$ =15,847, $N_{TD+info}$=375,842, N_{BD} =423,441 (**b**), or RC $N_{TD\text{-}info}$ =17,760, $N_{TD+info}$=373,929, N_{BD} =423,441 (**c**).

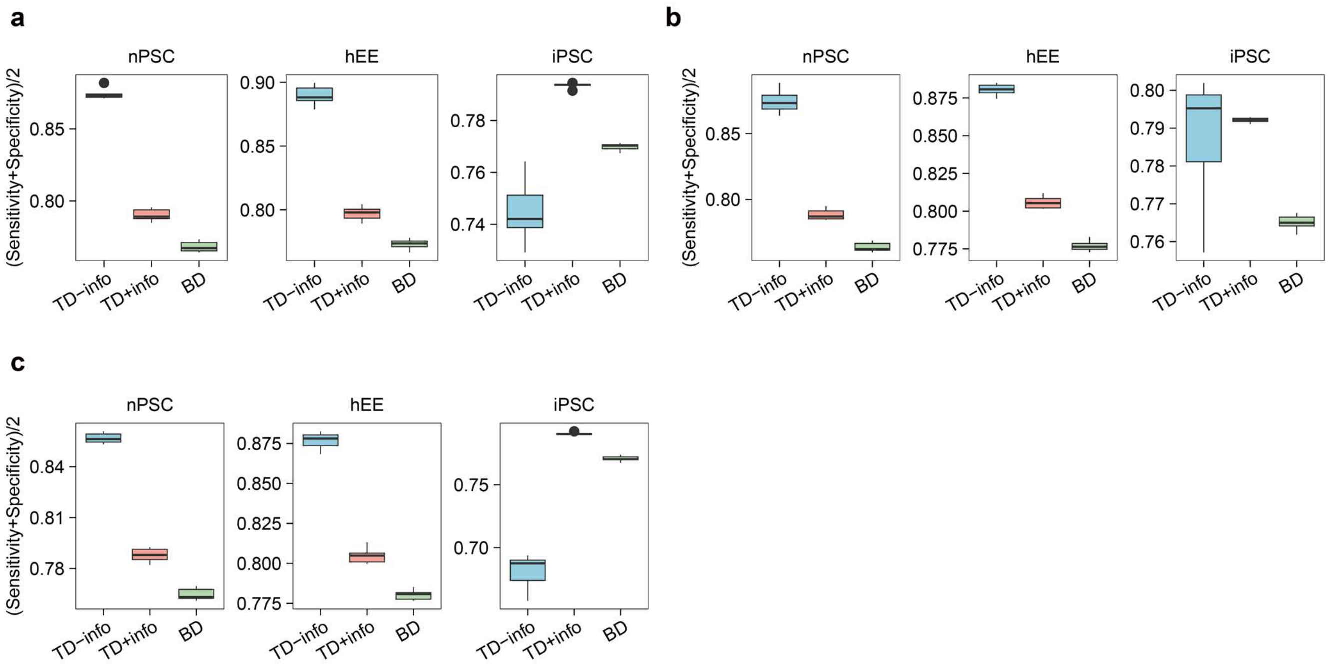

Supplementary Figure 35: The prediction accuracy of the scenario prediction model in nPSC, hEE and iPSC datasets. Cell similarities are estimated by RBP (**a**), PSI (**b**), and RC (**c**). The accuracy is estimated by the average of sensitivity and specificity. All boxes indicate median (center), Q25, and Q75 (bounds of box), the smallest value within 1.5 times interquartile range below Q25 and the largest value within 1.5 times interquartile range above Q75 (whiskers).

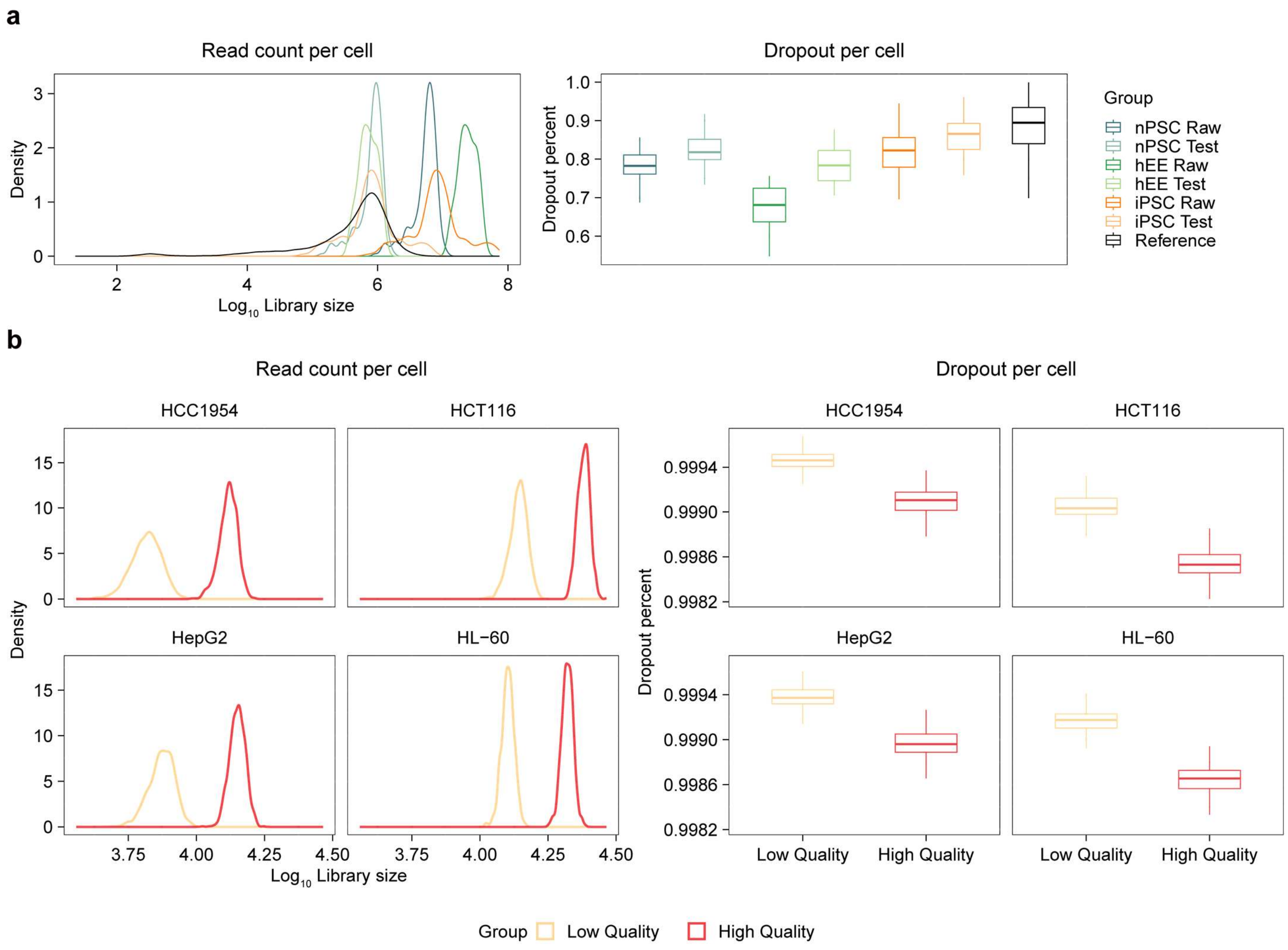

Supplementary Figure 36: Data quality evaluation across down-sampling and synthetic datasets. (**a**) The data qualities of nPSC, hEE and iPSC datasets. N_{nPSC}=225, N_{hEE}=90 and N_{iPSC}=174. The reference dataset consisted of more than 53k cell from 20 mouse organs[24]. left panel: density plot of library size. right panel: box plot of dropout rate in cells. (b). The data qualities of synthetic datasets. left panel: density plot of library size. right panel: box plot of dropout rate in cells. Each cell line generated 1000 cells for both the low-quality and high-quality groups. All boxes indicate median (center), Q25, and Q75 (bounds of box), the smallest value within 1.5 times interquartile range below Q25 and the largest value within 1.5 times interquartile range above Q75 (whiskers).

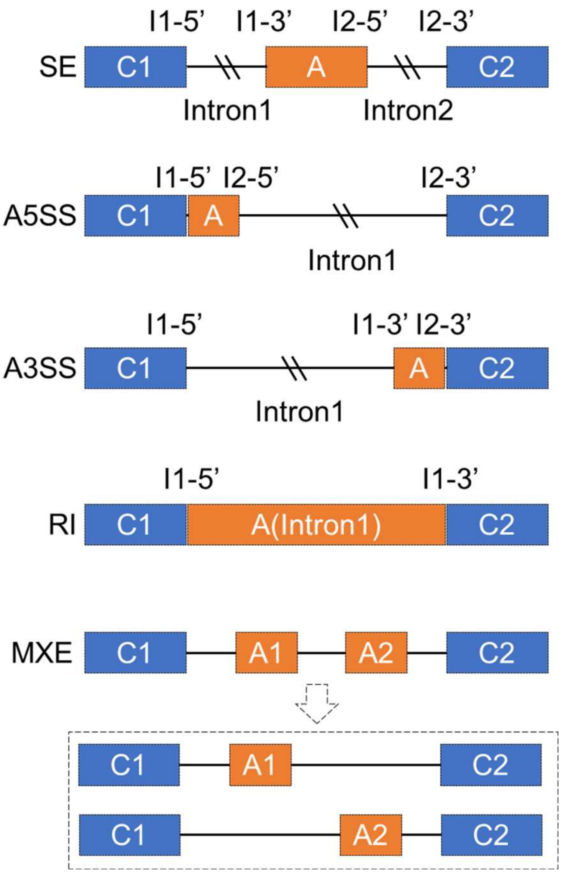

Supplementary Figure 37: Region definition in different AS types. I1-5' and I1-3' indicates the 5' and 3' splicing site of intron 1, respectively. I2-5' and I2-3' indicates the 5' and 3' splicing site of intron 2, respectively. C1 and C2 represent the two exon regions and A represent the alternative region.

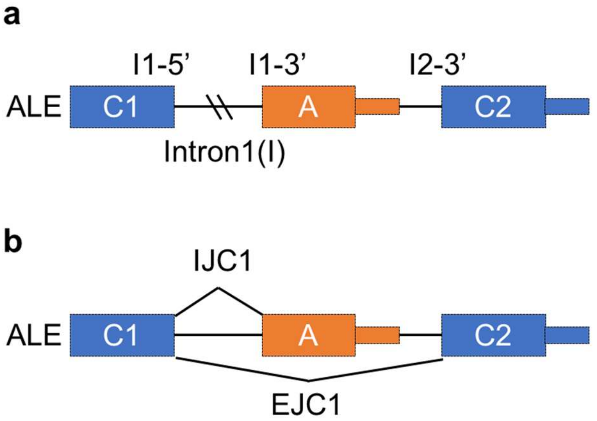

$$PSI_{A3SS} = \frac{IJC1}{IJC1 + EJC1}$$

Supplementary Figure 38: The event structure (**a**) and PSI definition (**b**) of ALE events.

Reference

1 Pollard, K. S., Hubisz, M. J., Rosenbloom, K. R. & Siepel, A. Detection of nonneutral substitution rates on mammalian phylogenies. *Genome Res* **20**, 110-121, doi:10.1101/gr.097857.109 (2010).

2 Trapnell, C. *et al.* Transcript assembly and quantification by RNA-Seq reveals unannotated transcripts and isoform switching during cell differentiation. *Nat Biotechnol* **28**, 511-515, doi:10.1038/nbt.1621 (2010).

3 Alamancos, G. P., Pages, A., Trincado, J. L., Bellora, N. & Eyras, E. Leveraging transcript quantification for fast computation of alternative splicing profiles. *RNA* **21**, 1521-1531, doi:10.1261/rna.051557.115 (2015).

4 Zappia, L., Phipson, B. & Oshlack, A. Splatter: simulation of single-cell RNA sequencing data. *Genome Biol* **18**, 174, doi:10.1186/s13059-017-1305-0 (2017).

5 Lebrigand, K., Magnone, V., Barbry, P. & Waldmann, R. High throughput error corrected Nanopore single cell transcriptome sequencing. *Nature communications* **11**, 4025 (2020).

6 Li, H. Minimap2: pairwise alignment for nucleotide sequences. *Bioinformatics* **34**, 3094-3100 (2018).

7 Zheng, G. X. *et al.* Massively parallel digital transcriptional profiling of single cells. *Nature communications* **8**, 14049 (2017).

8 Cao, J. *et al.* The single-cell transcriptional landscape of mammalian organogenesis. *Nature* **566**, 496-502, doi:10.1038/s41586-019-0969-x (2019).

9 Gulati, G. S. *et al.* Single-cell transcriptional diversity is a hallmark of developmental potential. *Science* **367**, 405-411, doi:10.1126/science.aax0249 (2020).

10 Tabula Sapiens, C. *et al.* The Tabula Sapiens: A multiple-organ, single-cell transcriptomic atlas of humans. *Science* **376**, eabl4896, doi:10.1126/science.abl4896 (2022).

11 Peng, J. *et al.* Single-cell RNA-seq highlights intra-tumoral heterogeneity and malignant progression in pancreatic ductal adenocarcinoma. *Cell Res* **29**, 725-738, doi:10.1038/s41422-019-0195-y (2019).

12 Bergen, V., Lange, M., Peidli, S., Wolf, F. A. & Theis, F. J. Generalizing RNA velocity to transient cell states through dynamical modeling. *Nat Biotechnol* **38**, 1408-1414, doi:10.1038/s41587-020-0591-3 (2020).

13 Jang, J. S. *et al.* Molecular signatures of multiple myeloma progression through single cell RNA-Seq. *Blood Cancer J* **9**, 2, doi:10.1038/s41408-018-0160-x (2019).

14 Andreatta, M. & Carmona, S. J. UCell: Robust and scalable single-cell gene signature scoring. *Comput Struct Biotechnol J* **19**, 3796-3798, doi:10.1016/j.csbj.2021.06.043 (2021).

15 Needle, M. N. *et al.* The Multiple Myeloma Research Foundation (MMRF) CoMMpassSM Study: A Longitudinal Study in Newly-Diagnosed Multiple Myeloma Patients to Assess Genomic Profiles, Immunophenotypes and Clinical Outcomes. *Blood* **120**, 3980, doi:https://doi.org/10.1182/blood.V120.21.3980.3980 (2012).

16 Mulligan, G. *et al.* Gene expression profiling and correlation with outcome in clinical trials of the proteasome inhibitor bortezomib. *Blood* **109**, 3177-3188, doi:10.1182/blood-2006-09-044974 (2007).

17 Shi, L. *et al.* The MicroArray Quality Control (MAQC)-II study of common practices for the development and validation of microarray-based predictive models. *Nat Biotechnol* **28**, 827-838, doi:10.1038/nbt.1665 (2010).

18 Hanzelmann, S., Castelo, R. & Guinney, J. GSVA: gene set variation analysis for microarray and RNA-seq data. *BMC Bioinformatics* **14**, 7, doi:10.1186/1471-2105-14-7 (2013).

19 Andreatta, M. & Carmona, S. J. UCell: Robust and scalable single-cell gene signature scoring. *Computational and structural biotechnology journal* **19**, 3796-3798 (2021).

20 Chu, L. F. *et al.* Single-cell RNA-seq reveals novel regulators of human embryonic stem cell differentiation to definitive endoderm. *Genome Biol* **17**, 173, doi:10.1186/s13059-016-1033-x (2016).

21 Zhang, Y. *et al.* Definitive Endodermal Cells Supply an in vitro Source of Mesenchymal Stem/Stromal Cells. *Commun Biol* **6**, 476, doi:10.1038/s42003-023-04810-5 (2023).

22 Deng, K., Yao, J., Huang, J., Ding, Y. & Zuo, J. Abnormal alternative splicing promotes tumor resistance in targeted therapy and immunotherapy. *Transl Oncol* **14**, 101077, doi:10.1016/j.tranon.2021.101077 (2021).

23 Bradley, R. K. & Anczukow, O. RNA splicing dysregulation and the hallmarks of cancer. *Nat Rev Cancer* **23**, 135-155, doi:10.1038/s41568-022-00541-7 (2023).

24 Schaum, N. *et al.* Single-cell transcriptomics of 20 mouse organs creates a Tabula Muris: The Tabula Muris Consortium. *Nature* **562**, 367 (2018).
