# Supplementary material for: Deciphering splicing heterogeneity at single-cell resolution by SCSES
Source: Nat Commun. 2025 Oct 27;16:9459. doi: 10.1038/s41467-025-64517-5 (PMC12559242; doi:10.1038/s41467-025-64517-5)
Supplement: Supplementary file 2 — Reporting Summary [file 41467_2025_64517_MOESM2_ESM.pdf]

nature portfolio

Corresponding author(s): Zhaoqi Liu

Last updated by author(s): Sep 10, 2025

Reporting Summary

Nature Portfolio wishes to improve the reproducibility of the work that we publish. This form provides structure for consistency and transparency in reporting. For further information on Nature Portfolio policies, see our Editorial Policies and the Editorial Policy Checklist.

Statistics

For all statistical analyses, confirm that the following items are present in the figure legend, table legend, main text, or Methods section.

n/a	Confirmed	
[ ]	[x]	The exact sample size (*n*) for each experimental group/condition, given as a discrete number and unit of measurement
[ ]	[x]	A statement on whether measurements were taken from distinct samples or whether the same sample was measured repeatedly
[ ]	[x]	The statistical test(s) used AND whether they are one- or two-sided<br>*Only common tests should be described solely by name; describe more complex techniques in the Methods section.*
[x]	[ ]	A description of all covariates tested
[ ]	[x]	A description of any assumptions or corrections, such as tests of normality and adjustment for multiple comparisons
[ ]	[x]	A full description of the statistical parameters including central tendency (e.g. means) or other basic estimates (e.g. regression coefficient) AND variation (e.g. standard deviation) or associated estimates of uncertainty (e.g. confidence intervals)
[ ]	[x]	For null hypothesis testing, the test statistic (e.g. *F*, *t*, *r*) with confidence intervals, effect sizes, degrees of freedom and *P* value noted<br>*Give P values as exact values whenever suitable.*
[x]	[ ]	For Bayesian analysis, information on the choice of priors and Markov chain Monte Carlo settings
[x]	[ ]	For hierarchical and complex designs, identification of the appropriate level for tests and full reporting of outcomes
[ ]	[x]	Estimates of effect sizes (e.g. Cohen's *d*, Pearson's *r*), indicating how they were calculated

Our web collection on statistics for biologists contains articles on many of the points above.

Software and code

Policy information about availability of computer code

Data collection	All raw fastq files of scRNA-seq data were downloaded with fastq-dump (v3.0.10) and wget (v1.21).
Data analysis	The implementation of SCSES is available at https://github.com/lvxuan12/SCSES. SCSES was developed in R 4.3.1 mainly with following pakage dependencies: shiny(v1.9.1), jsonlite(v1.8.9), rtracklayer(v1.58.0), Matrix(v1.6-5), reticulate(v1.39.0), BSgenome(v1.66.3), R.matlab(v3.7.0), and glmnet(4.1-8), et al . SCSES also needs Python (v3.11), Matlab compiler runtime (v9.13), and JAVA (v17.0.10). Other softwares used in SCSES include: STAR (v2.7.11a), samtools (v1.20), rMATS (v4.3.0), MAJIQ (v2.5.7), IRFinder (v1.3.0), et al. Other tools used in present analysis include Seurat (R package, v5.1.0), monocle3 (R package, v1.3.7), UCell (R package, v2.2.0), CytoTRACE2 (R package, v1.0.0), infercnv (R package v1.14.2), clusterProfiler(R package, v4.6.2), gProfiler(R package, v0.2.3),scvelo (Python package v0.3.2).

For manuscripts utilizing custom algorithms or software that are central to the research but not yet described in published literature, software must be made available to editors and reviewers. We strongly encourage code deposition in a community repository (e.g. GitHub). See the Nature Portfolio guidelines for submitting code & software for further information.

Data

Policy information about availability of data

All manuscripts must include a data availability statement. This statement should provide the following information, where applicable:

- Accession codes, unique identifiers, or web links for publicly available datasets
- A description of any restrictions on data availability
- For clinical datasets or third party data, please ensure that the statement adheres to our policy

All datasets analyzed in this study are publicly available. The bulk RNA-seq data of cell lines were downloaded from CCLE Project from NCBI Sequence Read Archive (SRA) under accession codes SRP186687. The single-cell RNA-seq data HCC1954 and HL60 were downloaded from NCBI SRA under accession codes SRP041736. The single-cell RNA-seq data of HCT116 and HepG2 were downloaded from Gene Expression Omnibus (GEO) under accession codes GSE150993. The paired long-read and short-read of ovarian cancer dataset was downloaded from SRA under accession PRJNA993664. The paired long-read and short-read of human hippocampus dataset was downloaded from The Neuroscience Multi-omic Data Archive (NeMO) under identifier nemo:dat-ho986e6 and nemo:dat-unjyo0u, respectively. The single-cell RNA-seq data of human naïve pluripotent stem cells, human early embryos, and induced human pluripotent stem cells were downloaded from GEO under accession codes GSE171820, GSE36552 and GSE85908, respectively. The cell-cycle synchronized datasets used for removing cell-cycle-dependent splicing events were downloaded from GEO under accession codes GSE123958, GSE81485, GSE143275, GSE216497, GSE97774, GSE94479, GSE116131 and PRJEB7566. The single-cell RNA-seq data of multiple myeloma patients were downloaded from GEO under accession codes GSE110499. The single-cell RNA-seq dataset for validation of multiple myeloma were downloaded from GEO under accession codes GSE118900, GSE9782, GSE24080 and TCGA MMRF project. The single-cell RNA-seq data of H9 human ESC differentiation were downloaded from GEO under accession codes GSE75748. The inDrop-v3 RNA-seq data of mouse hematopoietic stem cell were provided by Caleb Weinreb from Allon Klein Lab in Department of Systems Biology, Harvard Medical School, which can be accessed by Dropbox repository. Source data are provided with this paper.

Research involving human participants, their data, or biological material

Policy information about studies with human participants or human data. See also policy information about sex, gender (identity/presentation), and sexual orientation and race, ethnicity and racism.

Reporting on sex and gender	There are no human research participants.
Reporting on race, ethnicity, or other socially relevant groupings	There are no human research participants.
Population characteristics	There are no human research participants.
Recruitment	There are no human research participants.
Ethics oversight	There are no human research participants.

Note that full information on the approval of the study protocol must also be provided in the manuscript.

Field-specific reporting

Please select the one below that is the best fit for your research. If you are not sure, read the appropriate sections before making your selection.

☒ Life sciences ☐ Behavioural & social sciences ☐ Ecological, evolutionary & environmental sciences

For a reference copy of the document with all sections, see nature.com/documents/nr-reporting-summary-flat.pdf

Life sciences study design

All studies must disclose on these points even when the disclosure is negative.

Sample size	No additional method was used to predetermine sample size. The sample size depended on the availability of datasets, except for the difference in data preprocessing. For each analysis, the sample size was sufficient to derive statistically meaningful results.
Data exclusions	(1) In multiple myeloma dataset, to focus on the cells with BTZ resistance, cells from patient MM16 were excluded in analysis.<br>(2) In indrop dataset, to remove cells with low quality, cells with less than 500 expressed genes or library size less than 1000 were removed.
Replication	We validated our method on simulation dataset of four cell lines and three different down-sampling datasets (iPSC, hEE and nPSC). All tests were repeated for three times, and average values were taken for comparison.
Randomization	When data, environment and parameters are consistent, the imputation results are basically consistent.
Blinding	All results are based on published data which have been studied in their original publications. Therefore, blinding from investigators is not possible when we reanalyzed the data. Group allocation information was never provided to the computational algorithms.

Reporting for specific materials, systems and methods

We require information from authors about some types of materials, experimental systems and methods used in many studies. Here, indicate whether each material, system or method listed is relevant to your study. If you are not sure if a list item applies to your research, read the appropriate section before selecting a response.

Materials & experimental systems

n/a	Involved in the study
☒	☐ Antibodies
☒	☐ Eukaryotic cell lines
☒	☐ Palaeontology and archaeology
☒	☐ Animals and other organisms
☒	☐ Clinical data
☒	☐ Dual use research of concern
☒	☐ Plants

Methods

n/a	Involved in the study
☒	☐ ChIP-seq
☒	☐ Flow cytometry
☒	☐ MRI-based neuroimaging

Plants

Seed stocks	*Report on the source of all seed stocks or other plant material used. If applicable, state the seed stock centre and catalogue number. If plant specimens were collected from the field, describe the collection location, date and sampling procedures.*
Novel plant genotypes	*Describe the methods by which all novel plant genotypes were produced. This includes those generated by transgenic approaches, gene editing, chemical/radiation-based mutagenesis and hybridization. For transgenic lines, describe the transformation method, the number of independent lines analyzed and the generation upon which experiments were performed. For gene-edited lines, describe the editor used, the endogenous sequence targeted for editing, the targeting guide RNA sequence (if applicable) and how the editor was applied.*
Authentication	*Describe any authentication procedures for each seed stock used or novel genotype generated. Describe any experiments used to assess the effect of a mutation and, where applicable, how potential secondary effects (e.g. second site T-DNA insertions, mosiacism, off-target gene editing) were examined.*
