# Supplementary material for: Deciphering splicing heterogeneity at single-cell resolution by SCSES
Source: Nat Commun. 2025 Oct 27;16:9459. doi: 10.1038/s41467-025-64517-5 (PMC12559242; doi:10.1038/s41467-025-64517-5)
Supplement: Supplementary file 17 — Description of Additional Supplementary Files [file 41467_2025_64517_MOESM17_ESM.pdf]

Supplementary Data 1: Brief summary of AS quantification algorithms.

Supplementary Data 2: Event counts used for different comparison and different cells in cell line dataset.

Supplementary Data 3: Event information including genomic coordinates, gene names, and detection algorithms in the ovarian cancer dataset.

Supplementary Data 4: Event information including genomic coordinates, gene names, and detection algorithms in the human hippocampus dataset.

Supplementary Data 5: Evaluation Summary of Differentially splicing event detection.

Supplementary Data 6: 248 DSEs from genes without expression changes between SC1 and SC2 clusters.

Supplementary Data 7: 27 DSEs from genes reported to be associated with BTZ-resistance.

Supplementary Data 8: The AS marker events at each time points in hES dataset.

Supplementary Data 9: The correlation between DSEs and differentially expressed RBPs in hES dataset.

Supplementary Data 10: The AS marker events of each monocyte subgroup in HSC dataset.

Supplementary Data 11: Event features of A3SS, A5SS and RI events to calculate event similarity network.

Supplementary Data 12: Features used to predict event-cell pairs of BD and TD+Info.

Supplementary Data 13: Datasets used to identify splicing events exhibiting cell cycle-dependent regulation.

Supplementary Data 14: Event features of ALE events to calculate event similarity network.
